# Libertellenone C attenuates oxidative stress and neuroinflammation with the capacity of NLRP3 inhibition

**DOI:** 10.1007/s13659-024-00438-y

**Published:** 2024-02-26

**Authors:** Jie Cao, Lanqin Li, Runge Zhang, Zhou Shu, Yaxin Zhang, Weiguang Sun, Yonghui Zhang, Zhengxi Hu

**Affiliations:** 1 Department of Pharmacy, Union Hospital, Tongji Medical College, Huazhong University of Science and Technology, Wuhan, 430022 China; 2 Hubei Key Laboratory of Natural Medicinal Chemistry and Resource Evaluation, Tongji Medical College, Huazhong University of Science and Technology, Wuhan, 430030 Hubei China

**Keywords:** Neurodegenerative diseases, Narural products, Libertellenone C, NLRP3 inflammasome

## Abstract

Neurodegenerative diseases (NDs) are common chronic diseases arising from progressive damage to the nervous system. Here, in-house natural product database screening revealed that libertellenone C (LC) obtained from the fermentation products of *Arthrinium arundinis* separated from the gut of a centipede collected in our Tongji campus, showed a remarkable neuroprotective effect. Further investigation was conducted to clarify the specific mechanism. LC dose-dependently reversed glutamate-induced decreased viability, accumulated reactive oxygen species, mitochondrial membrane potential loss, and apoptosis in SH-SY5Y cells. Network pharmacology analysis predicted that the targets of LC were most likely directly related to oxidative stress and the regulation of inflammatory factor-associated signaling pathways. Further study demonstrated that LC attenuated nitrite, TNF-α, and IL-1β production and decreased inducible nitric oxide synthase and cyclooxygenase expression in lipopolysaccharide-induced BV-2 cells. LC could directly inhibit NLRP3 inflammasome activation by decreasing the expression levels of NLRP3, ASC, cleaved Caspase-1, and NF-κB p65. Our results provide a new understanding of how LC inhibits the NLRP3 inflammasome in microglia, providing neuroprotection. These findings might guide the development of effective LC-based therapeutic strategies for NDs.

**Supplementary Information:**

The online version contains supplementary material available at 10.1007/s13659-024-00438-y.

## Introduction

Neurodegenerative diseases (NDs) are late-onset diseases characterized by slow progressive damage to neuronal cells and neuronal loss, disrupting patients’ motor function, cognitive function, and homeostasis [1]. The incidence of NDs has increased as the population ages worldwide, prompting increased research attention. In the coming decades, NDs may become a major economic burden in healthcare [2]. Despite extensive research, the etiology of NDs has not yet been fully elucidated, and there are no truly effective therapeutic methods [3]. However, oxidative stress (OS) and neuroinflammation are thought to play an important role in ND pathogenesis, including Alzheimer’s disease (AD), Parkinson’s disease (PD), amyotrophic lateral sclerosis (ALS), and multiple sclerosis (MS) [4].

Neuroinflammation could be triggered during ND pathogenesis by the deposition of abnormal conformational proteins, signals from damaged neurons, or the imbalance between pro- and anti-inflammatory processes [5]. Microglial activation is the principal component of neuroinflammation in the central nervous system (CNS), and activated microglia usually polarize into two distinct phenotypes, the classical M1 and alternative M2 [6]. M1 microglia release proinflammatory factors, such as interleukin 1 beta (IL-1β), tumor necrosis factor-alpha (TNF-α), and inducible nitric oxide synthase (iNOS). In contrast, M2 microglia release anti-inflammatory factors, including CD206 and arginase-1 [7, 8]. Meanwhile, OS is a state in which reactive oxygen species (ROS) generation exceeds the capacity of the cellular antioxidant defense biosystem. Under normal conditions, cells counteract OS by modifying their homeostatic balance [9–11]. Excessive intracellular ROS production generally occurs during mitochondrial damage [12]. In general, the level of OS increases with age, and OS is considered an important factor that renders neuronal systems more vulnerable to multiple NDs, including AD and PD [13, 14]. Therefore, reducing neuroinflammation and OS may be the key to preventing postoperative neurological complications.

The inflammasome is a large polymeric protein complex and an important machine that triggers the inflammatory response [15]. The NF-κB signaling pathway is activated before inflammasome activation under the stimulation of LPS, which increases NLRP3 transcription and expression levels [16]. Inflammasome activation is triggered when the cellular NLRP3 level significantly increases, and a proteolytic cascade is initiated after NLRP3 inflammasome assembly. Under the action of Caspase-1, pro-IL-1β and pro-IL-18 are hydrolyzed to IL-1β and IL-18, which have proinflammatory activity [17]. This process leads to programmed cell death in the form of inflammation, namely, pyroptosis [18]. The CNS is prone to inflammation, and the activation of its inflammatory bodies is mainly mediated by microglia. The accumulation of inflammatory factors and the increase in damaged or dead cells cause tissue damage, and the increase in blood-brain barrier permeability seriously interferes with normal CNS functioning, demonstrating that the activation of inflammatory body NLRP3 is closely related to the induction and progression of AD [19, 20].

Natural products have interesting properties as complex molecules and are widely recognized as excellent sources for target drug candidate discovery [21]. 46% of drugs approved by the Food and Drug Administration from 1981 to 2014 are derived from natural small molecules. Many natural products, such as phenylpropanoids, terpenoids, flavonoids, and alkaloids, have shown neuroprotective effects in ND [22]. Therefore, it is critical to discover medicinal compounds that can remove oxygen-derived free radicals, improve mitochondrial function, and exert anti-inflammatory effects.

Libertellenone C (LC) was isolated from the fermentation products of the fungus *A. arundinis* obtained from the gut of a centipede collected in our Tongji campus. Pimarane diterpenes are important tricyclic diterpenes mainly distributed in higher plants, fungi, and marine organisms and show multiple biological activities, including anti-inflammatory effect [23]. Hence, in this study, we explored candidates with neuroprotective characteristics from an in-house compound library to obtain effective drug candidates for NDs, and our results provide a new understanding of the neuroprotective role of LC, which occurs via NLRP3 inflammasome inhibition in microglia.

## Results and discussion

### Neuroprotective effect of LC in SH-SY5Y cells exposed to H_2_O_2_ or glutamate

**Fig. 1:**
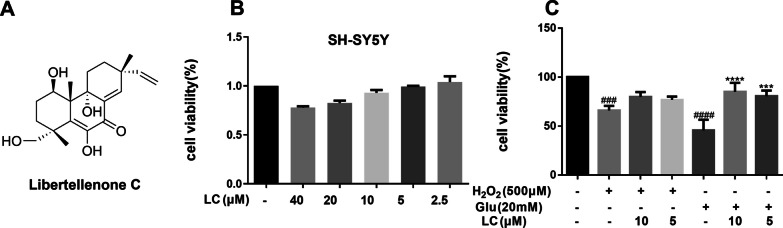
LC improved cell viability in H_2_O_2_- or glutamate-induced SH-SY5Y cell damage. **A** The chemical structure of LC. **B** LC showed no cytotoxic effect at concentrations up to 40 µM. **C** LC can protect SH-SY5Y cells from H_2_O_2_ (500 µM)- or glutamate (20 mM)-induced injury. Data are expressed as the mean ± SEM (*n* = 3). Differences were evaluated by one-way ANOVA or Student’s t test. The significance level was set at 0.05. ^###^*p* < 0.001 vs. the control group. ***p < 0.001 and ****p < 0.0001 vs. the glutamate-exposed group

H_2_O_2_ or glutamate-induced SH-SY5Y cell damage was applied to explore the lead compound with potential neuroprotective activities from the in-house natural compound library. LC, a pimarane diterpene isolated from the fungus *A. arundinis*, showed a remarkable neuroprotective effect and safety profile (Fig. 1, Additional file 1: Figs. S1–S4). LC demonstrated substantial protective effects against 20 mM glutamate-induced cell damage with an EC_50_ value of 4.98 µM and increased the viability of 500 µM H_2_O_2_-treated cells to 76.3–78.2% from 62.5%. Thus, glutamate-induced SH-SY5Y cell injury was chosen to explore the protective effects of LC further.

**Fig. 2:**
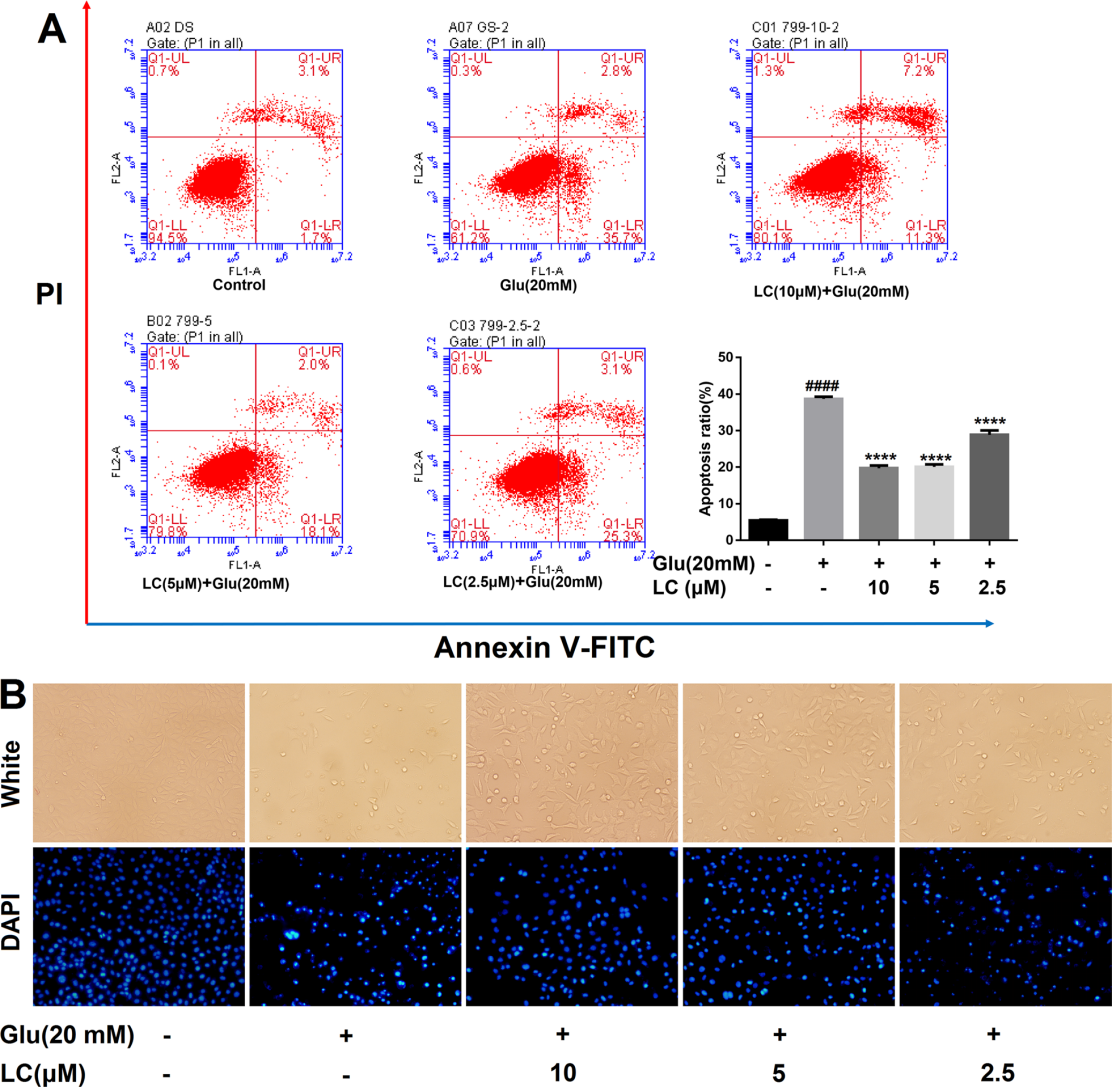
LC decreased apoptosis in SH-SY5Y cells exposed to glutamate. **A** Flow cytometry was applied to determine the apoptotic ratio after Annexin V-FITC/PI staining. The percentage of apoptotic cells is shown in the bar chart. **B** Cellular morphological changes were observed by phase contrast microscopy and DAPI staining. Data are expressed as the mean ± SEM (*n* = 3). Differences were evaluated by one-way ANOVA or Student’s t test. The significance level was set at 0.05. ^####^*P* < 0.0001 vs. the control group. ****P < 0.0001 vs. the glutamate group

**Fig. 3:**
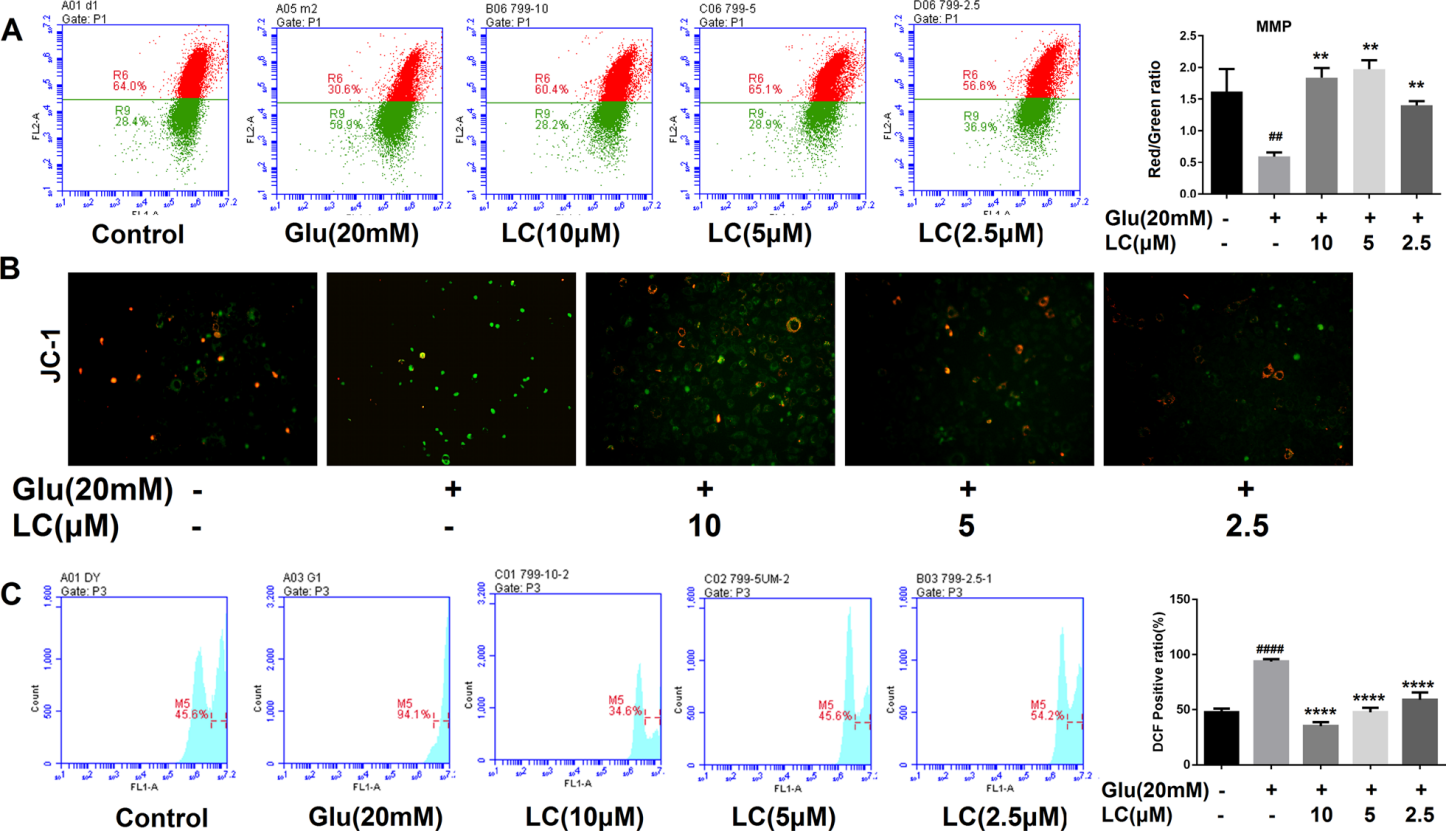
LC reduced the ROS level and relieved the decrease in MMP in SH-SY5Y cells induced with 20 mM glutamate. **A** JC-1 staining after LC treatment using a flow cytometer. **B** JC-1 staining by inverted fluorescence microscopy. **C** LC can reduce the ROS level in SH-SY5Y cells. Data are expressed as the mean ± SEM (*n* = 3). Differences were evaluated by one-way ANOVA or Student’s t test. The significance level was set at 0.05. ^####^*p* < 0.0001 vs. the control group. ****p < 0.0001 vs. the 20 mM glutamate group

**Fig. 4:**
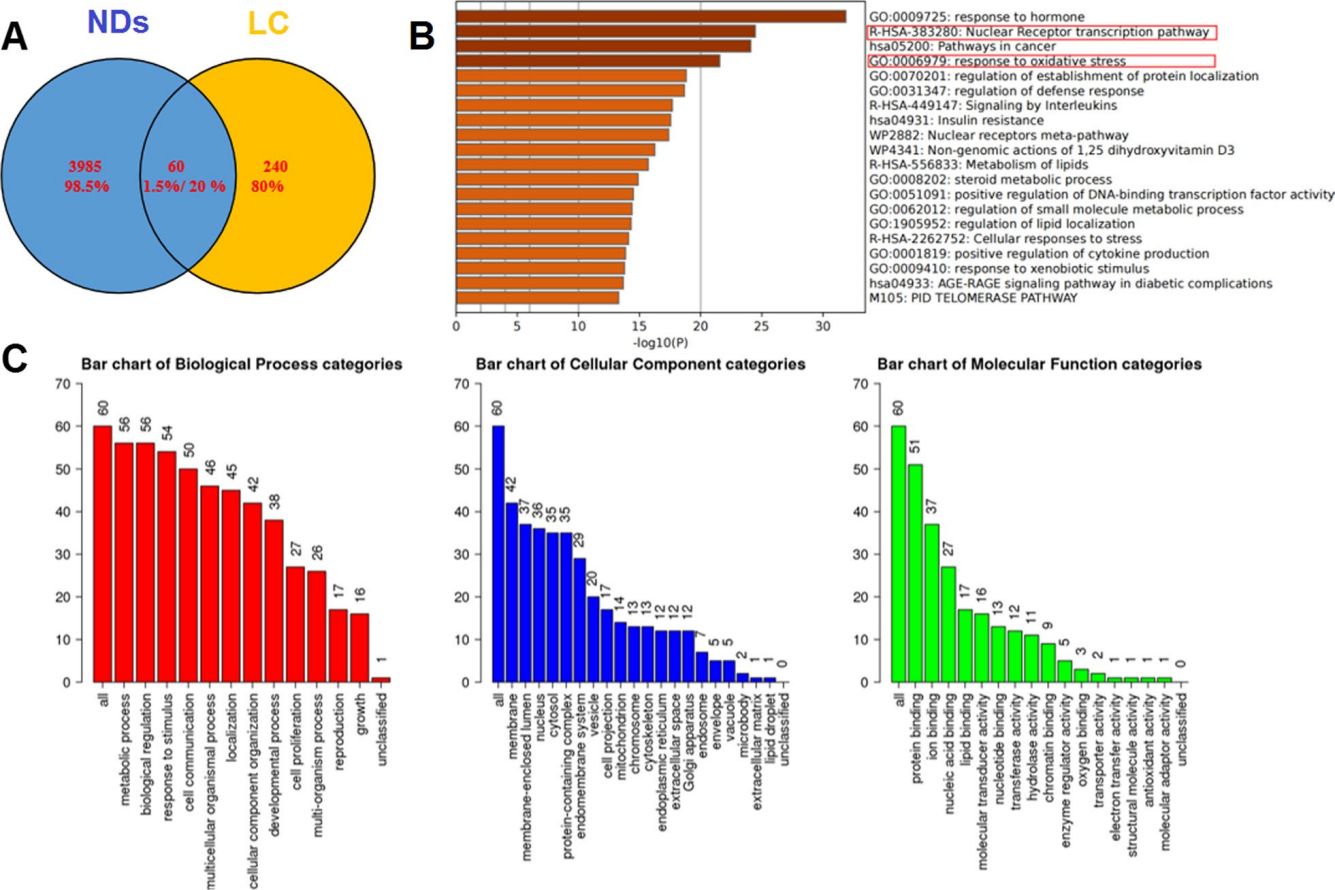
Bioinformatics analysis of potential drug targets of LC associated with neurodegenerative diseases. **A** The number of potential targets of LC in the treatment of neurodegenerative diseases. **B** Top 20 KEGG pathways of hub genes. **C** Gene ontology (GO) analysis of the associated genes using the WebGestalt tool

### Effects of LC on SH-SY5Y cell apoptosis after glutamate exposure

The Annexin V-FITC/PI double-staining method was used to further confirm the ability of LC to reduce apoptosis. Glutamate treatment dramatically increased the proportion of early apoptotic cells from 1.7 to 35.7%, while serial concentrations of LC (10, 5, and 2.5 µM) dose-dependently reduced the large proportion of early apoptotic cells to 11.3%, 18.1%, and 25.3%, respectively (Fig. 2A). DAPI stains the condensed nucleus of apoptotic cells bright blue; therefore, DAPI was used to detect the apoptotic features of glutamate-induced SH-SY5Y cells. The 20 mM glutamate sodium group contained few cells overall, and the stained apoptotic cells demonstrated irregular shapes, suggesting chromatin condensation and a shrunken nucleus, consistent with the white light observation images (Fig. 2B). Meanwhile, the cells possessed relatively normal morphology; the long shuttle type occupied a larger proportion in the LC-treated groups. These results demonstrate that LC can protect SH-SY5Y cells from apoptosis induced by 20 mM glutamate.

### Effects of LC on ROS production and mitochondrial dysfunction in SH-SY5Y cells exposed to glutamate

Excess intracellular ROS can induce mitochondrial depolarization and changes in mitochondrial membrane potential (MMP), which can aggravate cell apoptosis. JC-I is a fluorescent probe used extensively in the detection of MMP and exhibits potential-dependent accumulation in mitochondria. Glutamate (20 mM) reduced MMP, as demonstrated by the increased presence of the green fluorescent JC-1 monomer from 28.4 to 58.9%, while LC (10, 5, and 2.5 µM) reduced green fluorescence to 28.2%, 28.9%, and 36.95%, respectively (Fig. 3A). Simultaneously, compared to the normal group, 20 mM glutamate obviously increased the green fluorescence intensity, while cells treated with LC showed lower green fluorescence intensity and higher red fluorescence intensity than those in the Glu group, suggesting that LC can protect mitochondrial function (Fig. 3B).

The oxidation-sensitive fluorescent probe DCFH-DA was used to observe the free radical scavenging capacity to confirm the ability of LC to protect SH-SY5Y cells from glutamate-induced reactive stress. Glutamate markedly increased ROS levels from 45.6 to 94.1%, indicating an intense intracellular OS response. LC (10, 5, and 2.5 µM) reduced intracellular ROS levels from 94.1 to 34.6%, 45.6%, and 54.2%, respectively (Fig. 3C). These findings indicate that the neuroprotective activity of LC is partly related to its ability to reduce OS.

### Bioinformatics analysis of potential LC drug targets associated with NDs

Given the outstanding neuroprotective ability of LC, we analyzed its drug targets using pharmMapper to elucidate its specific target in NDs. The top 300 potential targets were predicted by the target confidence score (Additional file 1: Table S1). We compared 4045 gene targets related to NDs in the GeneCards and DisGeNET databases, and 60 co-gene targets were directly related to NDs (Fig. 4A). This result verified our previous experimental findings. Then, we analyzed the potential function of co-gene targets using the database for annotation, visualization, and integrated discovery (DAVID). Kyoto Encyclopedia of Genes and Genomes (KEGG) analysis showed that 138 signaling pathways were involved, and the top 20 important pathways were identified (Fig. 4B, C). The above targets were most likely directly related to OS and the regulation of inflammatory factor-associated signaling pathways. Therefore, LPS-induced inflammatory injury in BV-2 cells was evaluated for further activity assessments and related mechanism verification.

### Effects of LC on LPS-induced production of pro-inflammatory mediators and cytokines in BV-2 microglia

**Fig. 5:**
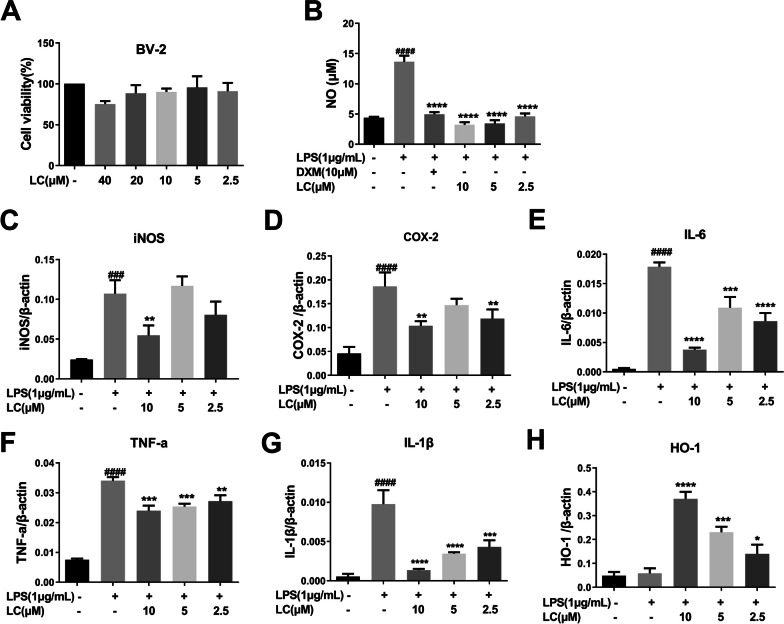
LC inhibited inflammatory proteins in LPS-induced BV-2 cells. **A** LC showed no toxicity toward BV-2 cells. **B** LC can inhibit NO generation in BV-2 cells as effectively as the positive control medicine dexamethasone (DXM 10 µM). **C**–**H** LC inhibited the expression of iNOS, HO-1, TNF-α, IL-1β, IL-6, and COX-2 in BV-2 cells. Data are expressed as the mean ± SEM (*n* = 3). Differences were evaluated by one-way ANOVA or Student’s t test. The significance level was set at 0.05. ^###^*p* < 0.001, ^####^*p* < 0.0001 vs. the control group. *p < 0.05, **p < 0.01, ***p < 0.001, ****p < 0.0001 vs. the LPS group

LC showed no toxicity toward BV-2 from 2.5 to 40 µM and obviously suppressed NO generation in BV-2 cells induced by LPS (1 µg/mL) (Fig. 5A-B). The number of proinflammatory mediators generated by activated microglia is critically related to neuronal degeneration and loss. To further verify the anti-neuroinflammatory effect of LC, we detected the mRNA levels of proinflammatory mediators by real-time quantitative PCR. LPS increased the levels of several inflammatory factors, such as TNF-α, IL-1β, and IL-6, and two inflammation-related enzymes, COX-2 and iNOS, in BV-2 cells; LC treatment inhibited the mRNA expression of these inflammatory factors (Fig. 5C-G). Meanwhile, LC increased the mRNA expression of HO-1, a classical antioxidative enzyme (Fig. 5H).

### Effects of LC on LPS-induced production of pro-inflammatory proteins in BV-2 microglia

**Fig. 6:**
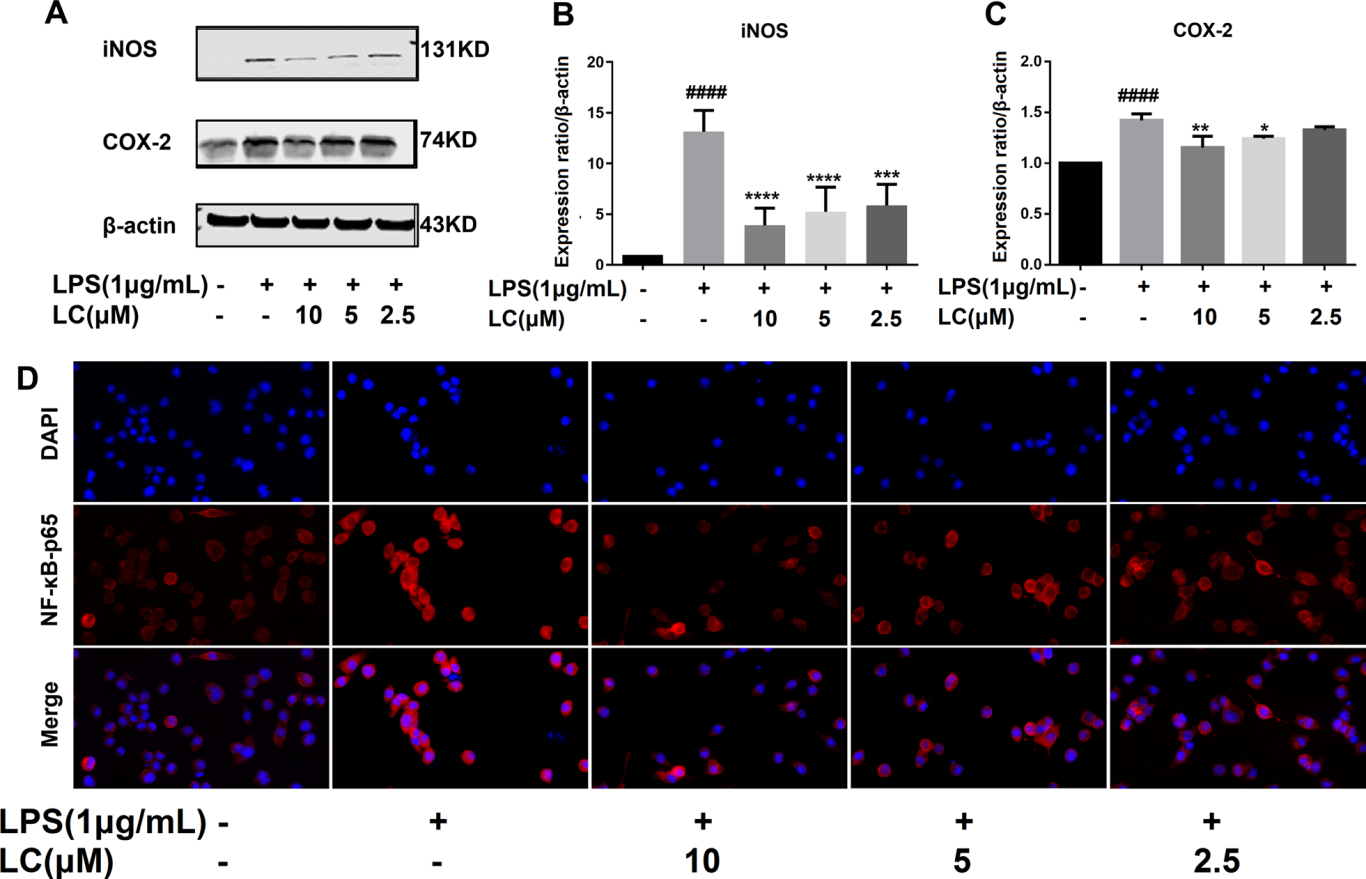
LC inhibited the expression of several inflammatory proteins in LPS-induced BV-2 cells. **A** Expression levels of COX-2 and iNOS. **B**, **C** COX-2 and iNOS band densities were quantified after normalization to the control band density. **D** Immunofluorescence results of NF-κB-P65. Data are expressed as the mean ± SEM (*n* = 3). Differences were evaluated by one-way ANOVA or Student’s t test. The significance level was set at 0.05. ^####^*p* < 0.0001 vs. the control group. *p < 0.05, **p < 0.01, ***p < 0.001, ****p < 0.0001 vs. the LPS group. β

LPS markedly increased the expression levels of COX-2 and iNOS, while LC (10, 5, and 2.5 µM) attenuated this effect (Fig. 6A–C). Immunofluorescence analysis was performed to further explore the effect of LC on the nuclear migration of the important inflammation-related protein NF-κB p65, and LC relieved the increased intensity of pink fluorescence induced by 1 µg/mL LPS. Thus, the experimental results demonstrate that LC can decrease the protein levels of proinflammatory mediators.

### Effects of LC on LPS-induced activation of NLRP3 inflammasome in BV-2 microglia

**Fig. 7:**
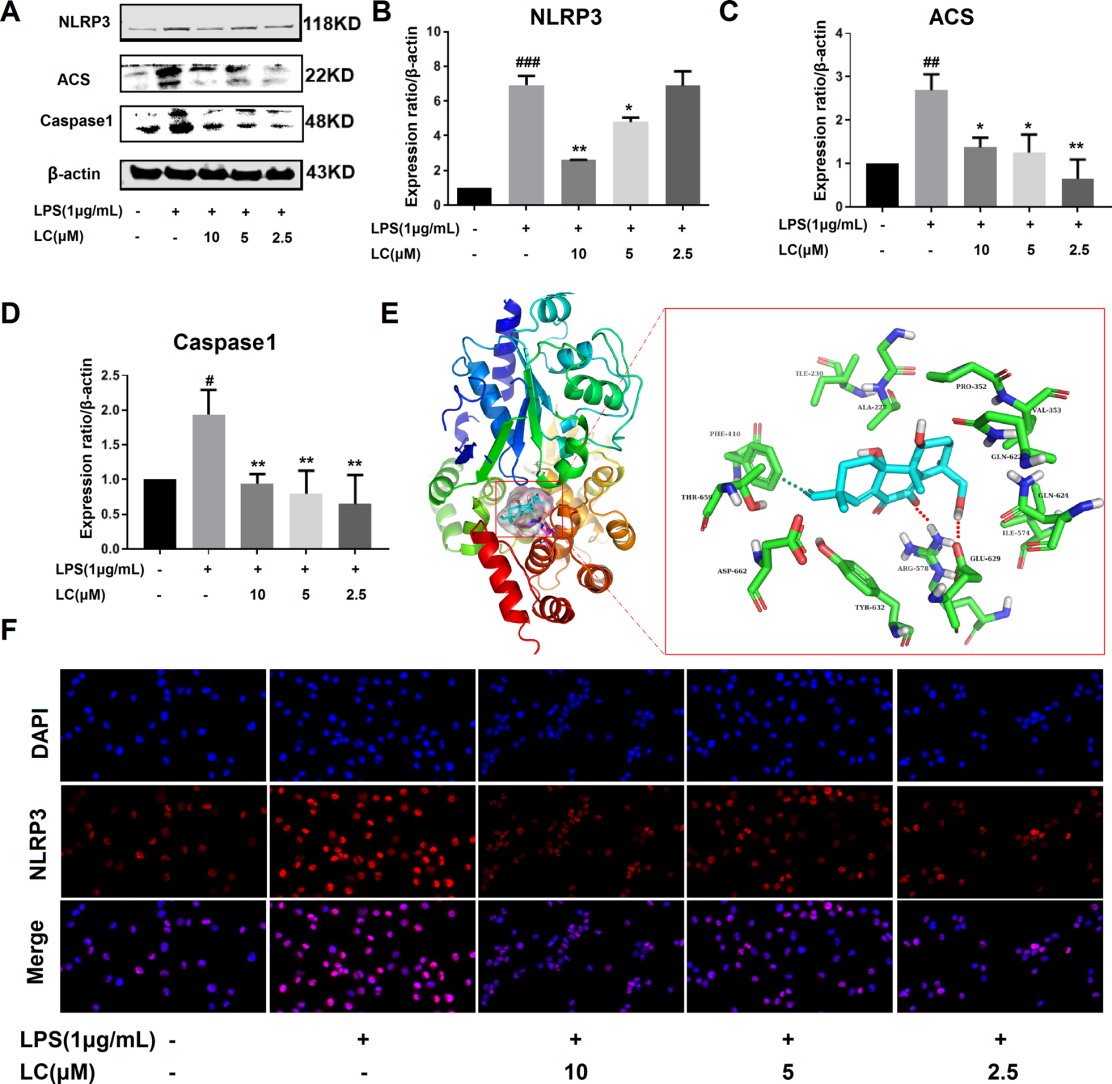
LC inhibited the activation of the NLRP3 inflammasome. **A** Expression levels of NLRP3, ASC, and Caspase-1. **B**–**D** NLRP3, ASC, and Caspase-1 band densities were quantified after normalization to the control band density. **E** The virtual docking model of LC with NLRP3. **F** Immunofluorescence results of NLRP3. Data are expressed as the mean ± SEM (*n* = 3). Differences were evaluated by one-way ANOVA or Student’s t test. The significance level was set at 0.05. ^###^*p* < 0.001, ^#^*p* < 0.05 vs. the control group. **p < 0.01, *p < 0.05 vs. the LPS group

The NLRP3 inflammasome pathway plays a vital role in inflammatory signaling pathways. The protein expression levels of NLRP3, Caspase-1, and ASC in BV-2 cells were measured by western blotting. LPS significantly increased the protein expression of NLRP3, ASC, and Caspase-1 (Fig. 7A–D). LC decreased the protein expression of NLRP3, ASC, and Caspase-1; however, regarding ASC and Caspase-1 expression, the effect was not concentration-dependent. Immunofluorescence analysis demonstrated that the LPS-treated cells were positive for NLRP3 (pink) in the cytoplasm, whereas control cells were not (Fig. 7F). NLRP3 expression was considerably lower in the LC-treated groups than in the LPS group. These results indicate that LC inhibits the inflammatory response, partly via the NLRP3-mediated inflammasome.

The inhibitory effect of LC on NLRP3 protein was most effective in the above experiment. Thus, molecular docking was further performed to investigate the binding mode of LC to the NLRP3 protein (PDB ID: 1NSI). LC was found to interact through hydrogen bonding with arginine at position 578 and glutamic acid at position 629 of NLRP3 (Fig. 7E). A π–π conjugated interaction with phenylalanine was also observed at position 410. In summary, LC is highly likely to inhibit NLRP3 assembly and activation by interacting with NLRP3, thus slowing the development of neuroinflammation and protecting neuronal cells.

### Effects of LC on the LPS-induced decline in autophagy and increase in oxidative stress levels in BV-2 microglia

**Fig. 8:**
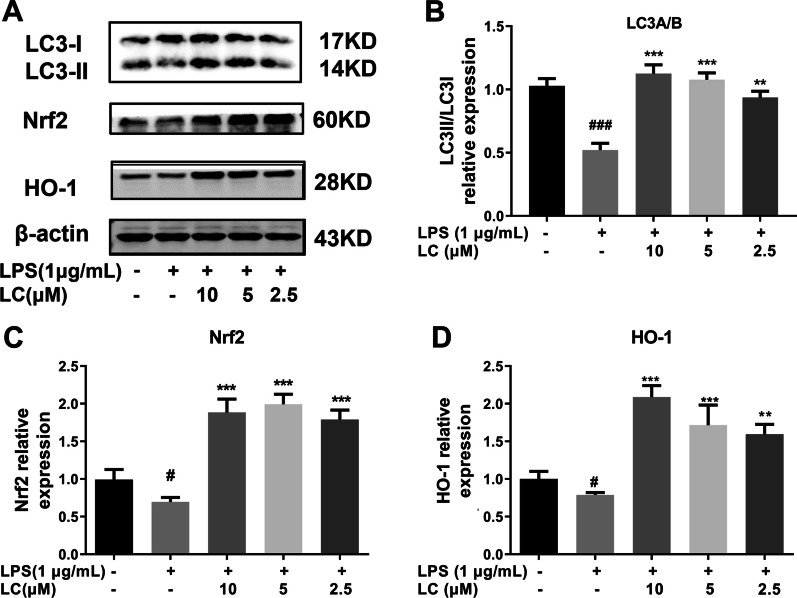
LC enhanced autophagy levels and inhibited oxidative stress in LPS-induced BV-2 cells. **A** Expression levels of LC3I, LC3II, Nrf2, and HO-1. **B** The LC3II band density was quantified after normalization to the LC3I band density. **C**, **D** Expression of Nrf2 and HO-1 density were quantified after normalization with control band density. Data are expressed as the mean ± SEM (*n* = 3). Differences were evaluated by one-way ANOVA or Student’s t test. The significance level was set at 0.05. ^###^*p* < 0.001 vs. the control group. ****p < 0.0001, ***p < 0.001, **p < 0.01, *p < 0.05 vs. the LPS group

We further measured how LPS-induced autophagy and OS regulate NLRP3 inflammasome activation in BV-2 cells (Fig. 8). Compared to the LPS group, LC obviously promoted the conversion of LC3-I into LC3-II (*p* < 0.05), implying that LC could downregulate the NLRP3 inflammasome and activate autophagy. LC also substantially increases HO-1 expression in BV-2 cells, manifesting as a significant increase in HO-1 mRNA and protein levels and the protein expression level of HO-1’s upstream regulatory protein Nrf2 (Fig. 8C, D). These results indicate that LC plays an antioxidant role through the Nrf2/HO-1 signaling pathway, further inhibiting the production of cellular oxygen free radicals and protecting neuronal cells.

## Discussion and conclusion

Glutamate is the most prevalent, widely distributed, and effective excitatory neurotransmitter in the CNS [24]. Neuronal toxicity occurs when the glutamate concentration in the intercellular space is too high, resulting in neuronal degeneration, aging, and death [25]. This excitotoxic effect of glutamate is closely related to the occurrence and development of various NDs and is one of the important mechanisms leading to neuronal cell death in NDs [26]. Human neuroblastoma cells (SH-SY5Y) with moderate dopamine β-hydroxylase activity are commonly used as a cell model of nerve injury [27]. Obvious increases in the apoptotic ratio, reactive oxygen species, and mitochondrial dysfunction were observed after culturing SH-SY5Y cells with 20 mM glutamate sodium for 24 h. Meanwhile, LC (10, 5, and 2.5 µM) can attenuate these insults, indicating that LC can reduce apoptotic cells, inhibit or scavenge excessive oxygen radicals, and protect the normal function of mitochondria.

Microglia are CNS immune cells that can protect neurons by phagocytosing pathogens and harmful particles in the brain tissue and can also activate reactive microglia that secrete inflammatory cytokines in response to inflammatory factors, resulting in neuronal toxicity [28, 29]. Therefore, reactive microglia are an important target for treating neuroinflammation and NDs [30]. LPS, a glycolipid composed of lipids and polysaccharides, is a constituent of the outer wall of gram-negative bacterial cell walls [31, 32]. LPS-induced microglial activation results in a series of inflammatory responses, such as the production of multiple proinflammatory mediators (ROS, RNS, PGE2, NO, iNOS, and COX-2) and cytokines (TNF-α, IL-6, and IL-1β), that further deteriorate neurons [33]. Damaged neurons produce many ROS, which activate microglia, thus forming a vicious cycle [34]. The NF-κB signal transduction pathway plays an important role in the inflammatory response [35–37]. Normally, IκB is confined to the cytoplasm when not activated and undergoes phosphorylation upon LPS stimulation. NF-κB is isolated from the IκB complex and continuously activated, translocating from the cytoplasm to the nucleus and binding to target gene promoters to activate transcription, leading to the synthesis and secretion of NO, TNF-α, and IL-6, generating inflammation [38]. LC can dramatically suppress NO generation as effectively as the positive medicine dexamethasone in BV-2 cells, preliminarily demonstrating its anti-neuroinflammatory function. RT-PCR, immunofluorescence, and western blotting techniques were applied to further explore LC’s mechanisms of action. LC was found to play an anti-inflammatory role by suppressing the NF-κB p65 signaling pathway and inhibiting the expression of cytokines, such as TNF-α, IL-1β, and IL-6, and several significant inflammatory factors, such as COX-2 and iNOS (Fig. 6). The activation of the NF-κB-P65/IκB pathway is often accompanied by the nuclear entry of NF-κB p65. Therefore, we performed immunofluorescence analysis to verify that LC could indeed inhibit the nuclear entry of NF-κB p65. This result was further confirmed by western blotting.

The NLRP3 inflammasome plays a significant role in neuroinflammation. This study demonstrated by immunofluorescence analysis that LC can indeed inhibit the activation of the NLRP3 inflammasome, and the results were further confirmed by western blotting. The NPAI Engine system was used to perform molecular docking between LC and NLRP3. The docking results also showed that LC closely binds to NLRP3 through hydrogen bonding, conjugation, hydrophobic interactions, and other intermolecular forces. Therefore, the natural product LC is highly likely to protect neuronal cells by inhibiting the activation of the NLRP3 inflammasome. Regarding the antioxidant mechanism of LC, we also found that different concentrations of LC could significantly increase the expression level of HO-1 in BV-2 cells, indicating potent antioxidant action.

In recent years, many studies have shown that autophagy plays an important role in regulating the inflammatory response [39]. Autophagy, an important way for the body to clear damaged or necrotic cells and organelles, is closely related to the occurrence and development of numerous human diseases [40]. Our study showed that 1 µg/mL LPS could decrease autophagy, while the autophagy level was relatively high in the LC and blank control groups. Thus, LC can enhance autophagy in BV-2 cells, contributing to the inactivation of the NLRP3 inflammasome, inhibiting the excessive inflammatory response, and playing a protective role. Therefore, LC is highly likely to exert an anti-neuroinflammatory effect by inhibiting NLRP3 inflammasome activation and enhancing autophagy levels.

In conclusion, LC plays a role in neuroprotection by resisting neuroinflammation, excessive OS, and mitochondrial dysfunction and reducing apoptotic cells by inhibiting the activation of the NLRP3 inflammasome. Therefore, LC is a promising candidate for ND prevention and treatment.

## Materials and methods

### Reagents

Libertellenone C (LC) was obtained as a white powder from the fermentation products of *A. arundinis*, which was analyzed by HR-MALDI mass spectrometry for the molecular formula C_20_H_28_O_5_ (obsd [M + Na]^+^ at *m*/*z* 371.1829, calcd [M + Na]^+^ 371.1834). LC was dissolved in DMSO (dimethyl sulfoxide, Sigma-Aldrich, MO, USA), with a stock concentration of 40 mM. H_2_O_2_ and sodium glutamate were purchased from RHAWN reagent (Shanghai, China). Antibodies against p-NF-κB p65 (SC-136548) and NF-κB p65 (SC-8008) were obtained from Santa Cruz Biotecnology, USA. Antibodies against NLRP3 (D2P5E, #13158), ASC (D2W8U, #67824), Caspase-1 (E2Z1C, #24232), iNOS (D6B6S, #13120), COX-2 (D5H5, #12282), HO-1 (E8B7A, #26416), and LC3A/B (D3U4C, #12741) were obtained from CST (Cell Signaling Technology, MA, USA). Antibodies against β-actin (Ab8226) were obtained from Abcam (Cambridge, UK).

### Cell culture

Human neuroblastoma SH-SY5Y and murine microglia BV-2 cell lines were purchased from Procell Life Science & Technology Co, Ltd (Wuhan, China). Cells were maintained in DMEM medium containing 10% FBS and 1% penicillin/streptomycin (Gibco, CA, USA) in a humidified incubator supplied with 95% air and 5% CO_2_ at 37 °C.

### Assays to determine potential neuroprotective activity

The cytotoxicity of the compounds was studied by the CCK-8 method (Biosharp Life Sciences, China). SH-SY5Y cells were seeded in 96-well plates (1 × 10^5^ cells/mL) for 24 h, the cells were treated with 40 µM of compounds. The concentration of DMSO was 0.1% of the medium culture were selected as control. After another 24 h treatment, the cell viability was measured with CCK-8 method. The cytotoxic values of > 40 µM were chosen for further analysis.

The neuroprotective activities of the compounds without toxicity were measured according to the published procedures with some modifications. SH-SY5Y cells were seeded into 96-well plates at a density of 1 × 10^5^ cells/mL. After 24 h of being incubated in the incubator, SH-SY5Y cells were pretreated with the test compounds for 4 h before incubation in a medium containing H_2_O_2_ (500 µM) or glutamate (20 mM). After another 24 h treatment, the cell viability was measured with CCK-8 method. Protection ratio % = (OD_test group_ – OD_model group_)/(OD_control group_ – OD_model group_) × 100%. The cells treated with H_2_O_2_ (500 µM) or glutamate (20 mM) were the model group, the cells treated with only 0.1% DMSO were selected as control group. The compounds with > 50% protection ratio were tested with series concentrations and the intergroup difference was compared by GraphPad Prism V8.0 (GraphPad Software, Inc., San Diego, CA).

### DAPI staining for detection of nuclear condensation

In this study, DAPI (Solarbio Life Sciences, China) was applied to analyze the apoptotic impact of the compounds on SH-SY5Y cells according to a previously described protocol. The cells were seeded into 12-well plates at a density of 5 × 10^5^ cells/well for 24 h. Then LC (10, 5, 2.5 µM) was co-incubated for 4 h and 20 mM glutamate sodium was added for another 24 h. Specifically, After fixing with 4% paraformaldehyde, the cells were washed with cold phosphate-buffered saline twice and incubated with DAPI staining solution at 37 °C for 20 min. After effective staining, next, wash three times to remove the superfluous DAPI staining solution was washed to avoid strong background, and an OLYMPUS IX73 fluorescence microscope (Olympus, Tokyo, Japan) was used to image the stained cells.

### Annexin V-FITC/PI staining for detection of apoptosis

Apoptosis was assessed using an Annexin V-FITC and propidium iodide (PI) detection kit (KGI Biotech, Nanjing, China) as described by the manufacturer’s instructions. A flowmeter (BD Acurri C6, China) was applied to analyze the apoptosis.

### Detection of intracellular reactive oxygen species (ROS)

After treatment as described above, the intracellular ROS production was measured using a non-fluorescent compound 2′,7′-dichlorofluorescein diacetate (DCFH-DA) (Solarbio Life Sciences, China) following the manufacturer’s instructions. Finally, a flowmeter (BD Acurri C6, China) was applied to analyze ROS production.

### Measurement of mitochondrial membrane potential (MMP)

The mitochondrial membrane potential (MMP) was measured with JC-1 mitochondrial membrane potential assay kit (Solarbio Life Sciences, China) following the manufacturer’s instructions. The flowmeter (BD Acurri C6, China) and an OLYMPUS IX73 fluorescence microscope (Olympus, Tokyo, Japan) were applied to analyze mitochondrial membrane potential.

### Network pharmacology analysis

The target genes of “Neurodegenerative diseases” were accessed from the GeneCards database (http://www.genecards.org/) and the DisGeNET database (https://www.disgenet.org/) [41, 42]. The possible drug targets of LC were obtained from Pharmapper database [43]. After removing duplicate targets, the common targets of both LC and Neurodegenerative diseases were obtained by using the Venny 2.1 (https://bioinfogp.cnb.csic.es/tools/venny/). The overlapped genes were the targets of this article that we researched. To study the biological function of potential targets in Neurodegenerative diseases, DAVID database (https://david.ncifcrf.gov/) was used to analysis Kyoto Encyclopedia of Genes and Genomes (KEGG) pathways [44]. And finally, KEGG data were uploaded to the Bioinformatics (http://www.bioinformatics.com.cn/) platform for visual analysis.

### Determination of nitric oxide content

BV-2 cells were seeded into 96-well plates at a density of 1 × 10^5^ cells/mL, after incubation of 24 h, cells were given compound LC (10, 5, 2.5 µM) and positive compound dexamethasone (10 µM) for 1 h, LPS (1 µg/mL) were added into corresponding wells. 24 h later, 50 µL supernatants were transferred into clean 96-well plates, following, the NO content in the supernatants was measured by Griess reaction according to the instruction of Nitric Oxide Assay Kit (Pulilai Gene Technology Co. LTD, Beijing, China). Finally, the absorbance was measured at 540 nm using a microplate reader (Varioskan LUX, 3020-80560). The concentration of nitrite was calculated according to the sodium nitrate standard curve.

### Immunofluorescence staining

Immunofluorescence staining was used to display the nuclear transfer of NF-κB p65 and the content change of NLRP3. BV-2 cells were seeded into 6-well plates containing cell crawling sheets at a density of 1.5 × 10^6^ cells/mL, after incubation of 24 h, and cells were given compound LC (10, 5, 2.5 µM), 1 h later, LPS (1 µg/mL) was added into corresponding groups. 24 h later, supernatants were removed, and cells were fixed with 4% paraformaldehyde for further analysis according to previous report. After blocking with 5% goat serum, the cells were incubated with NLRP3 antibody (1:1000, CST) and NF-κB p65 (1:1000, CST) overnight at 4 °C. Next, cells were incubated with Alexa Fluor 488-conjugated secondary goat anti-rabbit IgG antibody (1:5000, Abcam) at 37 °C for 1 h and DAPI (10 µg/mL) for 5 min. A fluorescence microscope (80i, Nikon Japan) was used to observe the results.

### Quantitative real-time PCR

Gene expression of BV-2 cells was analyzed by Quantitative RT-PCR (qRT-PCR). Total RNA was extracted using TRIzol reagent (Invitrogen, USA). The RNAs were reverse-transcribed into cDNA using a transcription kit (ABP, USA). qRT-PCR analysis was performed using SYBR Green qPCR Mix (ABP, USA) with 0.2 µM forward and reverse primers in a final volume of 10 µL, and detection was performed using ABI QuantStudio 5 (Thermo Fisher Scientific, USA). The resulting cDNA was amplified by incubating at 95 °C for 5 min, 40 cycles of denaturation at 95 °C for 10 s, annealing at 55–60 °C for 20 s, and extension at 72 °C for 30 s. Values were exhibited relative to β-actin. Each primer’s sequence was displayed as follows:

- HO-1 forward: 5′-TGGCATCTTCCCCAACGAAA-3′,
- HO-1 reverse: 5′- ATGGCCGTGTCAACAAGGAT-3′;
- COX-2 forward: 5′-AGAAGGAAATGGCTGCAGAA-3′,
- COX-2 reverse: 5′-GCTCGGCTTCCAGTATTGAG-3′;
- iNOS forward: 5′-TTGGCTCCAGCATGTACCCTC-3′,
- iNOS reverse: 5′-TGCTTCGGACATCAAAGGTCT-3′;
- TNF-α forward: 5′-CGTCGTAGCAAACCACCAAGT-3′,
- TNF-α reverse: 5′-CCATCGGCTGGCACCACTA-3′;
- IL-1β forward: 5′-CTACCTGTGTCTTTCCCGTG-3′,
- IL-1β reverse: 5′-TTTGTTGTTCATCTCGGAGC-3′;
- IL-6 forward: 5′-TAAAATAGTCCTTCCTACCCC-3′,
- IL-6 reverse: 5′-TTGCCGAGTAGATCTCAAA-3′;
- β-Actin forward: 5′-CGTGCGTGACATCAAAGAGAA-3′,
- β-Actin reverse: 5′-TGGATGCCACAGGATTCCAT-3′;

### Western blotting analysis

The expression levels of relative proteins were identified by western blotting. Total proteins from the BV-2 cells were lysed with RIPA lysis buffer mixed with PMSF protease inhibitor (Beyotime, China) and cocktails protease inhibitor (Beyotime, China) with a ratio of 100:1:1. Protein concentration was determined by BCA protein detection kit. The extracted proteins were separated by sodium-dodecylsulfate polyacrylamide gel electrophoresis (SDS-PAGE). The separated proteins are transferred from the gel to a nitrocellulose filter membrane (NC, Millipore, USA). The membranes were blocked in BSA (Beyotime, China) for 1 h and then incubated overnight with 1:1000 dilutions of anti-iNOS, anti-COX2, anti-NLRP3, anti-ASC, anti-Caspase1, anti-HO-1, anti-IκB, anti-LC3A/B, anti-NF-κB p65, anti-Nrf2, anti-Keap1. After incubation with the secondary antibody anti-mouse IgG (H+L) (DyLight™ 800, CST, USA) at 1:15000 dilutions, the membranes were imaged using a LiCor Odyssey scanner (LICOR, USA). Protein expressions were normalized using β-actin as the reference (CST, USA) in the same sample.

### Statistical analysis

All data are represented as means ± SEM for three independent trials performed in triplicate. Statistical analyses were performed by one-way ANOVA using Dunnett’s multiple comparison test, or Student’s t-test. *p* < 0.05 was considered as a statistically significant difference compared to controls.

### Supplementary Information

**Additional file 1: Figure S1.** The ^1^H NMR spectrum of libertellenone C. **Figure S2.** The ^13^C NMR spectrum of libertellenone C.**Figure S3.** The DEPT spectrum of libertellenone C. **Figure S4.** The spectroscopic data of libertellenone C. **Table S1. **Top 300 targets of LC predicted by pharMapper. **Figure S5.** Original western blot for three repeats.

## Data Availability

All data generated or analyzed during this study are available in this published article and its Additional files.

## References

[1] Qiao J, Wang T, Shao Z, Zhu Y, Zhang M, Huang S, Zeng P (2023). Genetic correlation and gene-based pleiotropy analysis for four major neurodegenerative diseases with summary statistics. Neurobiol Aging.

[2] Wilson DM, Cookson MR, Van Den Bosch L, Zetterberg H, Holtzman DM, Dewachter I (2023). Hallmarks of neurodegenerative diseases. Cell.

[3] Simon DK, Tanner CM, Brundin P (2020). Parkinson disease epidemiology, pathology, genetics, and pathophysiology. Clin Geriatr Med.

[4] Glass CK, Saijo K, Winner B, Marchetto MC, Gage FH (2010). Mechanisms underlying inflammation in neurodegeneration. Cell.

[5] Ransohoff RM (2016). How neuroinflammation contributes to neurodegeneration. Science.

[6] Colton CA (2009). Heterogeneity of microglial activation in the innate immune response in the brain. J Neuroimmune Pharmacol.

[7] Kwon HS, Koh SH (2020). Neuroinflammation in neurodegenerative disorders: the roles of microglia and astrocytes. Transl Neurodegener.

[8] Hong Y, Dong X, Chang L, Xie C, Chang M, Aguilar JS, Lin J, Lin J, Li QQ (2023). Microglia-containing cerebral organoids derived from induced pluripotent stem cells for the study of neurological diseases. iScience.

[9] El Khoury J (2010). Neurodegeneration and the neuroimmune system. Nat Med.

[10] Liddelow SA, Guttenplan KA, Clarke LE, Bennett FC, Bohlen CJ, Schirmer L, Bennett ML, Münch AE, Chung WS, Peterson TC, Wilton DK, Frouin A, Napier BA, Panicker N, Kumar M, Buckwalter MS, Rowitch DH, Dawson VL, Dawson TM, Stevens B, Barres BA (2017). Neurotoxic reactive astrocytes are induced by activated microglia. Nature.

[11] Nayak D, Roth TL, McGavern DB (2014). Microglia development and function. Annu Rev Immunol.

[12] Zhu T, Guo J, Wu Y, Lei T, Zhu J, Chen H, Kala S, Wong KF, Cheung CP, Huang X, Zhao X, Yang M, Sun L (2023). The mechanosensitive ion channel Piezo1 modulates the migration and immune response of microglia. iScience.

[13] Venigalla M, Sonego S, Gyengesi E, Sharman MJ, Münch G (2016). Novel promising therapeutics against chronic neuroinflammation and neurodegeneration in Alzheimer’s disease. Neurochem Int.

[14] Zhang WJ, Li KY, Lan Y, Zeng HY, Chen SQ, Wang H (2023). NLRP3 inflammasome: a key contributor to the inflammation formation. Food Chem Toxicol.

[15] Wang T, Xu H, Dong R, Wu S, Guo Y, Wang D (2023). Effectiveness of targeting the NLRP3 inflammasome by using natural polyphenols: a systematic review of implications on health effects. Food Res Int.

[16] Afonina IS, Zhong Z, Karin M, Beyaert R (2017). Limiting inflammation–the negative regulation of NF-κB and the NLRP3 inflammasome. Nat Immunol.

[17] Bauernfeind FG, Horvath G, Stutz A, Alnemri ES, MacDonald K, Speert D, Fernandes-Alnemri T, Wu J, Monks BG, Fitzgerald KA, Hornung V, Latz E (2009). Cutting edge: NF-κB activating pattern recognition and cytokine receptors license NLRP3 inflammasome activation by regulating NLRP3 expression. J Immunol.

[18] Simard JC, Cesaro A, Chapeton-Montes J, Tardif M, Antoine F, Girard D, Tessier PA (2013). S100A8 and S100A9 induce cytokine expression and regulate the NLRP3 inflammasome via ROS-dependent activation of NF-κB1. PLoS ONE.

[19] Thapa P, Upadhyay SP, Singh V, Boinpelly VC, Zhou J, Johnson DK, Gurung P, Lee ES, Sharma R, Sharma M (2023). Chalcone: a potential scaffold for NLRP3 inflammasome inhibitors. Eur J Med Chem Rep.

[20] Liu X, Zhang Z, Ruan J, Pan Y, Magupalli VG, Wu H, Lieberman J (2016). Inflammasome-activated gasdermin D causes pyroptosis by forming membrane pores. Nature.

[21] Porte Alcon S, Gorojod RM, Kotler ML (2020). Kinetic and protective role of autophagy in manganese-exposed BV-2 cells. Biochim Biophys Acta Mol Cell Res.

[22] Shao QH, Zhang XL, Chen Y, Zhu CG, Shi JG, Yuan Y, Chen NH (2018). Anti-neuroinflammatory effects of 20C from *Gastrodia elata* via regulating autophagy in LPS-activated BV-2 cells through MAPKs and TLR4/Akt/mTOR signaling pathways. Mol Immunol.

[23] Costantino V, Fattorusso E, Mangoni A, Perinu C, Cirino G, De Gruttola L, Roviezzo F (2009). Tedanol: a potent anti-inflflammatory *ent*-pimarane diterpene from the Caribbean Sponge *Tedania ignis*. Bioorg Med Chem.

[24] Maitra U, Stephen C, Ciesla LM (2022). Drug discovery from natural products—old problems and novel solutions for the treatment of neurodegenerative diseases. J Pharm Biomed Anal.

[25] Joyner PM, Cichewicz RH (2011). Bringing natural products into the fold—exploring the therapeutic lead potential of secondary metabolites for the treatment of protein-misfolding-related neurodegenerative diseases. Nat Prod Rep.

[26] Chib S, Singh S (2022). Manganese and related neurotoxic pathways: a potential therapeutic target in neurodegenerative diseases. Neurotoxicol Teratol.

[27] Javaid HMA, Ko E, Joo EJ, Kwon SH, Park JH, Shin S, Cho KW, Huh JY (2023). TNFα-induced NLRP3 inflammasome mediates adipocyte dysfunction and activates macrophages through adipocyte-derived lipocalin 2. Metabolism.

[28] Xu Z, Ji R, Zha X, Zhao H, Zhou S (2023). The aqueous extracts of *Ageratum conyzoides* inhibit inflammation by suppressing NLRP3 inflammasome activation. J Ethnopharmacol.

[29] Duan Y, Wang J, Cai J, Kelley N, He Y (2022). The leucine-rich repeat (LRR) domain of NLRP3 is required for NLRP3 inflammasome activation in macrophages. J Biol Chem.

[30] Singh L, Singh S (2023). Neuroprotective potential of Honokiol in ICV-STZ induced neuroinflammation, Aβ (1–42) and NF-kB expression in experimental model of rats. Neurosci Lett.

[31] Xu Z, Zhou X, Hong X, Wang S, Wei J, Huang J, Ji L, Yang Y, Efferth T, Hong C, Li C (2023). Essential oil of *Acorus tatarinowii* Schott inhibits neuroinflammation by suppressing NLRP3 inflammasome activation in 3× Tg-AD transgenic mice. Phytomedicine.

[32] Anderson FL, Biggs KE, Rankin BE, Havrda MC (2023). NLRP3 inflammasome in neurodegenerative disease. Transl Res.

[33] Tejera D, Mercan D, Sanchez-Caro JM, Hanan M, Greenberg D, Soreq H, Latz E, Golenbock D, Heneka MT (2019). Heneka, systemic inflammation impairs microglial Aβ clearance through NLRP3 inflammasome. EMBO J.

[34] Jayaraj RL, Azimullah S, Parekh KA, Ojha SK, Beiram R (2022). Effect of citronellol on oxidative stress, neuroinflammation and autophagy pathways in an in vivo model of Parkinson’s disease. Heliyon.

[35] Deng Z, Dong Y, Zhou X, Lu JH, Yue Z (2022). Pharmacological modulation of autophagy for Alzheimer’s disease therapy: opportunities and obstacles. Acta Pharm Sin B.

[36] Minchev D, Kazakova M, Sarafian V (2022). Neuroinflammation and autophagy in Parkinson’s disease—novel perspectives. Int J Mol Sci.

[37] Wang X, Feng L, Xin M, Hao Y, Wang X, Shang P, Zhao M, Hou S, Zhang Y, Xiao Y, Ma D, Feng J (2020). Mechanisms underlying astrocytic connexin-43 autophagy degradation during cerebral ischemia injury and the effect on neuroinflammation and cell apoptosis. Biomed Pharmacother.

[38] Cui M, Yoshimori T, Nakamura S (2022). Autophagy system as a potential therapeutic target for neurodegenerative diseases. Neurochem Int.

[39] Wang ZY, Liu J, Zhu Z, Su CF, Sreenivasmurthy SG, Iyaswamy A, Lu JH, Chen G, Song JX, Li M (2021). Traditional Chinese medicine compounds regulate autophagy for treating neurodegenerative disease: a mechanism review. Biomed Pharmacother.

[40] Schiøtz BL, Roos N, Rishovd AL, Gjøen T (2010). Formation of autophagosomes and redistribution of LC3 upon in vitro infection with infectious salmon anemia virus. Virus Res.

[41] Rebhan M, Chalifa-Caspi V, Prilusky J, Lancet D (1997). GeneCards: integrating information about genes, proteins and diseases. Trends Genet.

[42] Bauer-Mehren A, Rautschka M, Sanz F, Furlong LI (2010). DisGeNET: a cytoscape plugin to visualize, integrate, search and analyze gene-disease networks. Bioinformatics.

[43] Yuan Q, Zhang X, Wei W, Furlong LI (2022). Lycorine improves peripheral nerve function by promoting Schwann cell autophagy via AMPK pathway activation and MMP9 downregulation in diabetic peripheral neuropathy. Pharmacol Res.

[44] Huang DW, Sherman BT, Lempicki RA (2009). Systematic and integrative analysis of large gene lists using DAVID bioinformatics resources. Nat Protoc.

