# Supplementary material for: Libertellenone C attenuates oxidative stress and neuroinflammation with the capacity of NLRP3 inhibition
Source: Nat Prod Bioprospect. 2024 Feb 26;14(1):17. doi: 10.1007/s13659-024-00438-y (PMC10897105; doi:10.1007/s13659-024-00438-y)
Supplement: Supplementary file 1 — Additional file 1: Figure S1. The 1H NMR spectrum of libertellenone C. Figure S2. The 13C NMR spectrum of libertellenone C.Figure S3. The DEPT spectrum of libertellenone C. Figure S4. The spectroscopic data of libertellenone C. Table S1. Top 300 targets of LC predicted by pharMapper. Figure S5. Original western blot for three repeats. [file 13659_2024_438_MOESM1_ESM.docx]

**Libertellenone C attenuates oxidative stress and** **neuroinflammation with the capacity of NLRP3 inhibition**

Jie Cao^1,†^, Lanqin Li^2,†^, Runge Zhang^2,†^, Zhou Shu^1^, Yaxin Zhang^2^, Weiguang Sun^2,*^, Yonghui Zhang^2,*^, Zhengxi Hu^2,^^*^

^1^ Department of Pharmacy, Union Hospital, Tongji Medical College, Huazhong University of Science and Technology, Wuhan 430030, China.

^2^ Hubei Key Laboratory of Natural Medicinal Chemistry and Resource Evaluation, Tongji Medical College, Huazhong University of Science and Technology, Wuhan 430030, Hubei, China.

* Corresponding author: E-mail: [weiguang_sun@hust.edu.cn](mailto:weiguang_s@hust.edu.cn) (W. Sun); [zhangyh@mails.tjmu.edu.cn](mailto:zhangyh@mails.tjmu.edu.cn) (Y. Zhang); [huzhengxi@hust.edu.cn](mailto:huzhengxi@hust.edu.cn) (Z. Hu)

^†^ These authors contributed equally to this work.


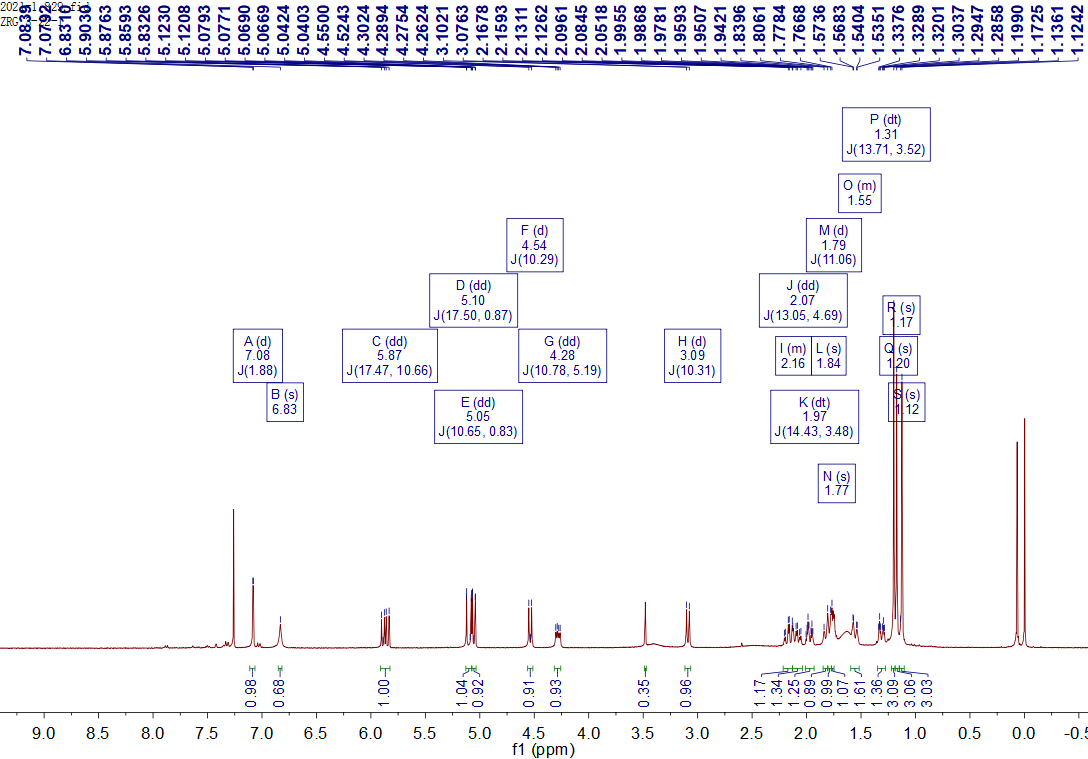
**Figure S1.** The ^1^H NMR spectrum of libertellenone C


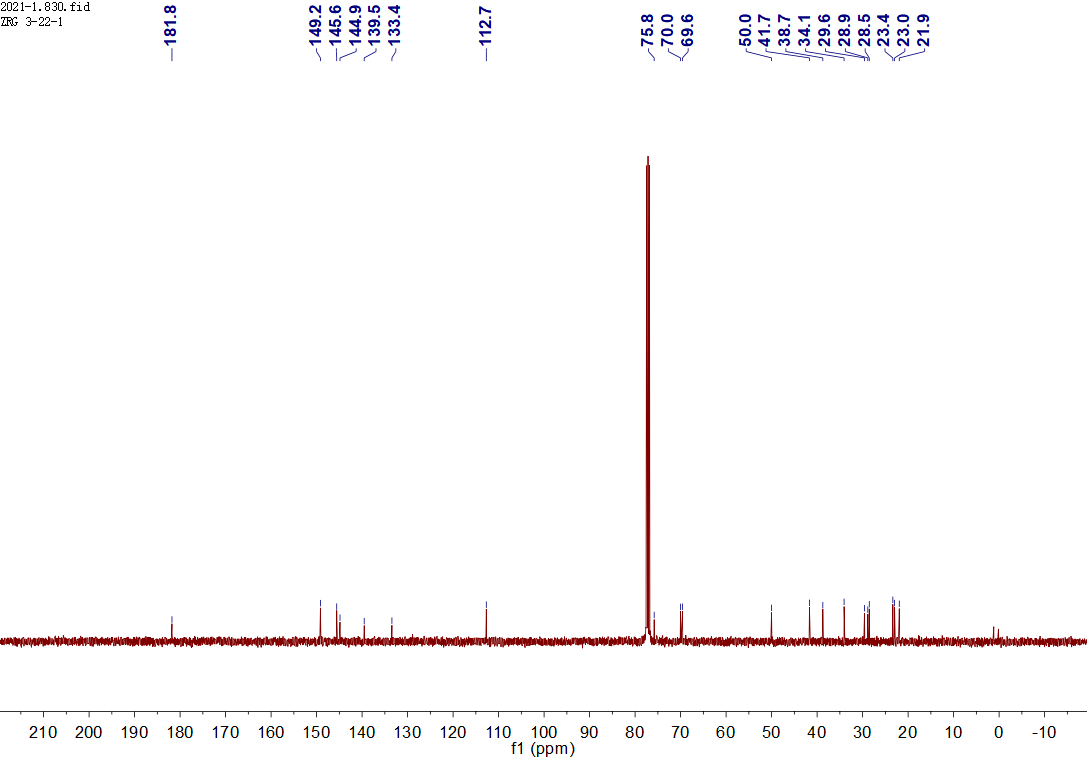


**Figure S2.** The ^13^C NMR spectrum of libertellenone C


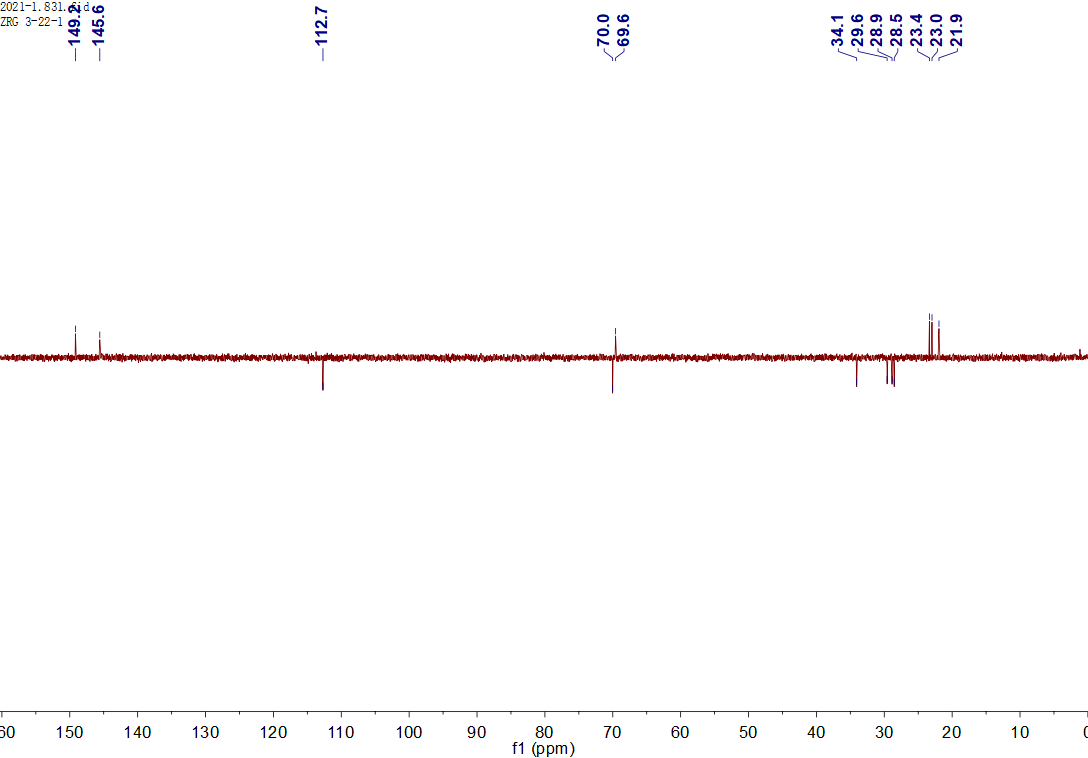


**Figure S3.** The DEPT spectrum of libertellenone C


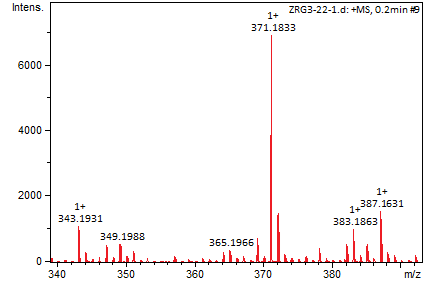


**Figure S4.** The spectroscopic data of libertellenone C

**Table S1.** Top 300 targets of LC predicted by pharMapper

|  | | | | | |
| --- | --- | --- | --- | --- | --- |
|  | PDB ID | Target Name | 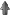Number of Features | 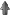Fit Score | 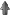Normalized Fit Score |
| 1 | 2HX1 | possible sugar phosphatase, HAD superfamily | 3 | 2.994 | 0.9981 |
| 2 | 3HZQ | Large-conductance mechanosensitive channel | 3 | 2.993 | 0.9975 |
| 3 | 2I1Y | Receptor-type tyrosine-protein phosphatase-like N | 3 | 2.99 | 0.9967 |
| 4 | 3CE6 | Adenosylhomocysteinase | 3 | 2.988 | 0.9961 |
| 5 | 1WH9 | 40S ribosomal protein S3 | 3 | 2.982 | 0.994 |
| 6 | 2NYA | Periplasmic nitrate reductase | 3 | 2.978 | 0.9927 |
| 7 | 1TU1 | Hypothetical protein | 3 | 2.972 | 0.9906 |
| 8 | 2ZM5 | tRNA Delta(2)-isopentenylpyrophosphate transferase | 3 | 2.968 | 0.9893 |
| 9 | 1GHH | DNA-damage-inducible protein I | 3 | 2.968 | 0.9893 |
| 10 | 2V3C | Signal recognition particle 19 kDa protein | 3 | 2.968 | 0.9893 |
| 11 | 2D6F | Glutamyl-tRNA(Gln) amidotransferase subunit D | 3 | 2.968 | 0.9892 |
| 12 | 1XK4 | Protein S100-A8 | 3 | 2.962 | 0.9875 |
| 13 | 1GT5 | Odorant-binding protein | 3 | 2.961 | 0.987 |
| 14 | 2KDD | Borealin | 3 | 2.957 | 0.9858 |
| 15 | 2P67 | LAO/AO transport system kinase | 3 | 2.954 | 0.9845 |
| 16 | 1T0T | UPF0447 protein GK3416 | 3 | 2.953 | 0.9842 |
| 17 | 2PIH | Uncharacterized protein ymcA | 3 | 2.95 | 0.9832 |
| 18 | 3EMJ | Inorganic pyrophosphatase | 4 | 3.476 | 0.8691 |
| 19 | 1RF8 | Eukaryotic translation initiation factor 4E | 4 | 3.193 | 0.7984 |
| 20 | [6NPY](https://www.rcsb.org/structure/6NPY) | NLRP3 inflammasome | 4 | 3.185 | 0.7765 |
| 21 | 2DQN | Glutamyl-tRNA(Gln) amidotransferase subunit A | 4 | 2.994 | 0.7485 |
| 22 | 2A2G | Major urinary protein | 4 | 2.993 | 0.7482 |
| 23 | 2OV6 | V-type ATP synthase subunit F | 4 | 2.991 | 0.7477 |
| 24 | 3BXZ | Protein translocase subunit secA | 4 | 2.991 | 0.7476 |
| 25 | 1XIO | Bacteriorhodopsin | 4 | 2.99 | 0.7474 |
| 26 | 1F5X | Proto-oncogene vav | 4 | 2.988 | 0.7471 |
| 27 | 3CT4 | PTS-dependent dihydroxyacetone kinase, dihydroxyacetone-binding subunit dhaK | 4 | 2.987 | 0.7467 |
| 28 | 8API | Cysteine proteinase 1, mitochondrial | 4 | 2.986 | 0.7466 |
| 29 | 1NVM | 4-hydroxy-2-oxovalerate aldolase | 4 | 2.986 | 0.7466 |
| 30 | 1N2S | dTDP-4-dehydrorhamnose reductase | 4 | 2.986 | 0.7465 |
| 31 | 3HKI | Prothrombin | 4 | 2.985 | 0.7462 |
| 32 | 1FFT | Ubiquinol oxidase subunit 1 | 4 | 2.983 | 0.7458 |
| 33 | 2YXR | Preprotein translocase subunit secY | 4 | 2.983 | 0.7458 |
| 34 | 1R6R | Genome polyprotein | 4 | 2.981 | 0.7453 |
| 35 | 2Q2E | Type II DNA topoisomerase VI subunit A | 4 | 2.98 | 0.7451 |
| 36 | 1FJ4 | 3-oxoacyl-[acyl-carrier-protein] synthase 1 | 4 | 2.979 | 0.7447 |
| 37 | 1ES8 | Type II restriction enzyme BglII | 4 | 2.976 | 0.744 |
| 38 | 2OPX | Lactaldehyde dehydrogenase | 4 | 2.976 | 0.744 |
| 39 | 1YAA | Aspartate aminotransferase, cytoplasmic | 4 | 2.976 | 0.7439 |
| 40 | 1UD9 | DNA polymerase sliding clamp A | 4 | 2.975 | 0.7437 |
| 41 | 2GFP | Multidrug resistance protein D | 4 | 2.975 | 0.7437 |
| 42 | 2VRE | Delta(3,5)-Delta(2,4)-dienoyl-CoA isomerase, mitochondrial | 4 | 2.974 | 0.7435 |
| 43 | 1IEB | H-2 class II histocompatibility antigen, E-D alpha chain | 4 | 2.973 | 0.7433 |
| 44 | 2JHQ | Uracil-DNA glycosylase | 4 | 2.973 | 0.7433 |
| 45 | 1TAF | Transcription initiation factor TFIID subunit 9 | 4 | 2.971 | 0.7428 |
| 46 | 1N8Z | Receptor tyrosine-protein kinase erbB-2 | 4 | 2.969 | 0.7424 |
| 47 | 1GMG | Regulatory protein rop | 4 | 2.969 | 0.7423 |
| 48 | 3BHY | Death-associated protein kinase 3 | 4 | 2.968 | 0.742 |
| 49 | 1TYB | Tyrosyl-tRNA synthetase | 4 | 2.968 | 0.7419 |
| 50 | 2PO0 | Probable exosome complex exonuclease 1 | 4 | 2.968 | 0.7419 |
| 51 | 3B8C | ATPase 2, plasma membrane-type | 4 | 2.967 | 0.7417 |
| 52 | 2NRO | Molybdopterin biosynthesis protein moeA | 4 | 2.965 | 0.7413 |
| 53 | 1QRJ | Gag-Pro-Pol polyprotein | 4 | 2.962 | 0.7405 |
| 54 | 1YED | Ig gamma-1 chain C region secreted form | 4 | 2.961 | 0.7403 |
| 55 | 2HGK | Uncharacterized protein yqcC | 4 | 2.96 | 0.7399 |
| 56 | 2IBJ | Cytochrome b5 | 4 | 2.959 | 0.7397 |
| 57 | 2OWI | Regulator of G-protein signaling 18 | 4 | 2.958 | 0.7395 |
| 58 | 1OSA | Calmodulin | 4 | 2.958 | 0.7395 |
| 59 | 1XKE | E3 SUMO-protein ligase RanBP2 | 4 | 2.957 | 0.7394 |
| 60 | 1S7Q | H-2 class I histocompatibility antigen, K-B alpha chain | 4 | 2.955 | 0.7387 |
| 61 | 1FRV | Periplasmic [NiFe] hydrogenase small subunit | 4 | 2.954 | 0.7385 |
| 62 | 3DXR | Mitochondrial import inner membrane translocase subunit TIM9 | 4 | 2.953 | 0.7381 |
| 63 | 2RFO | Nucleoporin NIC96 | 4 | 2.951 | 0.7378 |
| 64 | 2I7K | Bromodomain-containing protein 7 | 4 | 2.95 | 0.7376 |
| 65 | 2VLD | UPF0286 protein PYRAB01260 | 4 | 2.949 | 0.7372 |
| 66 | 1IRZ | Two-component response regulator ARR10 | 4 | 2.946 | 0.7366 |
| 67 | 2HZ7 | Glutaminyl-tRNA synthetase | 4 | 2.945 | 0.7363 |
| 68 | 2AJ7 | Flagellar assembly factor fliW | 4 | 2.943 | 0.7358 |
| 69 | 1EVY | Glycerol-3-phosphate dehydrogenase [NAD+], glycosomal | 4 | 2.941 | 0.7352 |
| 70 | 2BTY | Acetylglutamate kinase | 5 | 3.567 | 0.7134 |
| 71 | 2G18 | Phycocyanobili | 5 | 3.447 | 0.6893 |
| 72 | 3FQD | 5-3 exoribonuclease 2 | 5 | 3.445 | 0.689 |
| 73 | 3CRW | ATP-dependent DNA helicase Saci_0192 | 5 | 3.431 | 0.6862 |
| 74 | 1ZTE | Superoxide dismutase [Mn], mitochondrial | 5 | 3.412 | 0.6824 |
| 75 | 3CH6 | Corticosteroid 11-beta-dehydrogenase isozyme 1 | 5 | 3.285 | 0.6569 |
| 76 | 1XWR | Regulatory protein CII | 5 | 3.268 | 0.6536 |
| 77 | 1X65 | Cold shock domain-containing protein E1 | 6 | 3.899 | 0.6498 |
| 78 | 1ZAG | Zinc-alpha-2-glycoprotein | 6 | 3.885 | 0.6476 |
| 79 | 3D3L | Arachidonate 12-lipoxygenase, 12S-type | 5 | 3.21 | 0.642 |
| 80 | 2RM4 | Enhancer of mRNA-decapping protein 3 | 5 | 3.196 | 0.6393 |
| 81 | 1TM0 | Uncharacterized protein BMEI1586 | 5 | 3.173 | 0.6346 |
| 82 | 1N6B | Cytochrome P450 2C5 | 5 | 3.159 | 0.6318 |
| 83 | 2W62 | 1,3-beta-glucanosyltransferase GAS2 | 5 | 3.15 | 0.6301 |
| 84 | 2P1Q | SKP1-like protein 1A | 5 | 3.147 | 0.6294 |
| 85 | 1L6S | Delta-aminolevulinic acid dehydratase | 6 | 3.759 | 0.6264 |
| 86 | 1SO0 | Aldose 1-epimerase | 5 | 3.108 | 0.6215 |
| 87 | 1WRU | Baseplate protein | 6 | 3.706 | 0.6177 |
| 88 | 2OHV | Glutamate racemase | 6 | 3.702 | 0.6169 |
| 89 | 2NQ2 | Probable ABC transporter permease protein HI1471 | 5 | 3.082 | 0.6164 |
| 90 | 1J3X | High mobility group protein B2 | 6 | 3.645 | 0.6075 |
| 91 | 2HMN | Naphthalene 1,2-dioxygenase subunit alpha | 5 | 3 | 0.6 |
| 92 | 3BZ6 | UPF0502 protein PSPTO_2686 | 5 | 3 | 0.6 |
| 93 | 1XJV | Protection of telomeres protein 1 | 5 | 3 | 0.6 |
| 94 | 3C2O | Nicotinate-nucleotide pyrophosphorylase [carboxylating] | 5 | 2.997 | 0.5995 |
| 95 | 2GJ3 | Nitrogen fixation regulatory protein | 5 | 2.994 | 0.5988 |
| 96 | 2FBW | Succinate dehydrogenase [ubiquinone] flavoprotein subunit, mitochondrial | 5 | 2.993 | 0.5987 |
| 97 | 2PFF | Fatty acid synthase subunit alpha | 6 | 3.589 | 0.5981 |
| 98 | 2H08 | Ribose-phosphate pyrophosphokinase 1 | 5 | 2.988 | 0.5975 |
| 99 | 1YB1 | Estradiol 17-beta-dehydrogenase 11 | 5 | 2.987 | 0.5973 |
| 100 | 1GGZ | Calmodulin-like protein 3 | 5 | 2.986 | 0.5973 |
| 101 | 3D47 | L-rhamnonate dehydratase | 5 | 2.986 | 0.5971 |
| 102 | 3G9K | Capsule biosynthesis protein capD | 5 | 2.985 | 0.597 |
| 103 | 1NY9 | HTH-type transcriptional activator tipA | 5 | 2.985 | 0.597 |
| 104 | 1OXY | Hemocyanin II | 5 | 2.985 | 0.5969 |
| 105 | 1IQ3 | RalBP1-associated Eps domain-containing protein 2 | 5 | 2.984 | 0.5969 |
| 106 | 2GKS | Probable bifunctional SAT/APS kinase | 5 | 2.983 | 0.5966 |
| 107 | 2E9Q | 11S globulin subunit beta | 5 | 2.982 | 0.5965 |
| 108 | 1BWY | Fatty acid-binding protein, heart | 5 | 2.982 | 0.5965 |
| 109 | 3DF0 | Calpain-2 catalytic subunit | 5 | 2.982 | 0.5963 |
| 110 | 3IBV | Exportin-T | 5 | 2.978 | 0.5956 |
| 111 | 1NSH | Protein S100-A11 | 5 | 2.978 | 0.5956 |
| 112 | 1YF6 | Reaction center protein H chain | 5 | 2.977 | 0.5954 |
| 113 | 2Q5E | Carboxy-terminal domain RNA polymerase II polypeptide A small phosphatase 2 | 5 | 2.977 | 0.5953 |
| 114 | 2D1Q | Luciferin 4-monooxygenase | 5 | 2.976 | 0.5953 |
| 115 | 2DK2 | Heterogeneous nuclear ribonucleoprotein R | 5 | 2.975 | 0.595 |
| 116 | 2QPO | Thymidine kinase | 5 | 2.975 | 0.595 |
| 117 | 1L0V | Fumarate reductase flavoprotein subunit | 5 | 2.975 | 0.5949 |
| 118 | 3B6X | General odorant-binding protein lush | 5 | 2.974 | 0.5948 |
| 119 | 1J55 | Protein S100-P | 5 | 2.973 | 0.5946 |
| 120 | 2J96 | Phycoerythrocyanin alpha chain | 5 | 2.972 | 0.5944 |
| 121 | 3BOA | Protein disulfide-isomerase | 5 | 2.972 | 0.5943 |
| 122 | 1PJS | Siroheme synthase | 5 | 2.971 | 0.5942 |
| 123 | 1NA6 | Type-2 restriction enzyme EcoRII | 5 | 2.969 | 0.5937 |
| 124 | 1NTG | Tyrosyl-tRNA synthetase, cytoplasmic | 7 | 4.155 | 0.5936 |
| 125 | 1WEX | Heterogeneous nuclear ribonucleoprotein L-like | 5 | 2.968 | 0.5936 |
| 126 | 1B7A | Phosphatidylethanolamine-binding protein 1 | 5 | 2.968 | 0.5935 |
| 127 | 2ES0 | Regulator of G-protein signaling 6 | 5 | 2.967 | 0.5934 |
| 128 | 2F8N | Histone H3.2 | 5 | 2.966 | 0.5931 |
| 129 | 1B0A | Bifunctional protein folD | 5 | 2.965 | 0.5931 |
| 130 | 3GH8 | Iodotyrosine dehalogenase 1 | 5 | 2.965 | 0.593 |
| 131 | 1BMV | RNA2 polyprotein | 5 | 2.963 | 0.5927 |
| 132 | 3ESW | Peptide-N(4)-(N-acetyl-beta-glucosaminyl)asparagine amidase | 5 | 2.963 | 0.5927 |
| 133 | 2PN7 | Gamma-glutamylcyclotransferase | 5 | 2.962 | 0.5925 |
| 134 | 1PUL | TPPP family protein C32E8.3 | 5 | 2.96 | 0.592 |
| 135 | 1ORD | Ornithine decarboxylase, inducible | 5 | 2.959 | 0.5919 |
| 136 | 1JK0 | Ribonucleoside-diphosphate reductase small chain 1 | 5 | 2.959 | 0.5919 |
| 137 | 1QXZ | Methionine aminopeptidase | 6 | 3.546 | 0.591 |
| 138 | 1QOX | Beta-glucosidase | 5 | 2.954 | 0.5907 |
| 139 | 1VRN | Photosynthetic reaction center cytochrome c subunit | 5 | 2.953 | 0.5907 |
| 140 | 1WP1 | Outer membrane protein oprM | 5 | 2.953 | 0.5906 |
| 141 | 1XES | Dihydropinosylvin synthase | 5 | 2.952 | 0.5905 |
| 142 | 1M9I | Annexin A6 | 5 | 2.949 | 0.5898 |
| 143 | 1AUR | Carboxylesterase 2 | 5 | 2.949 | 0.5898 |
| 144 | 1LRW | Methanol dehydrogenase subunit 1 | 6 | 3.538 | 0.5897 |
| 145 | 1KV9 | Quinohemoprotein alcohol dehydrogenase ADH IIB | 5 | 2.948 | 0.5895 |
| 146 | 1BUO | Zinc finger and BTB domain-containing protein 16 | 5 | 2.948 | 0.5895 |
| 147 | 1ODH | Chorion-specific transcription factor GCMa | 5 | 2.946 | 0.5892 |
| 148 | 2V95 | Corticosteroid-binding globulin | 6 | 3.533 | 0.5888 |
| 149 | 1ZP2 | RNA polymerase II holoenzyme cyclin-like subunit | 5 | 2.943 | 0.5886 |
| 150 | 2RHB | Replicase polyprotein 1ab | 5 | 2.942 | 0.5884 |
| 151 | 2AYN | Ubiquitin carboxyl-terminal hydrolase 14 | 5 | 2.942 | 0.5883 |
| 152 | 1A3X | Pyruvate kinase 1 | 5 | 2.941 | 0.5883 |
| 153 | 1V9S | Uracil phosphoribosyltransferase | 5 | 2.941 | 0.5882 |
| 154 | 3GWJ | Arylphorin | 6 | 3.528 | 0.5881 |
| 155 | 3EUH | Chromosome partition protein mukF | 6 | 3.501 | 0.5835 |
| 156 | 3EPM | Thiamine biosynthesis protein thiC | 6 | 3.499 | 0.5832 |
| 157 | 3CF3 | Transitional endoplasmic reticulum ATPase | 6 | 3.495 | 0.5824 |
| 158 | 2NQB | Histone H3 | 6 | 3.434 | 0.5723 |
| 159 | 1QGK | Importin subunit beta-1 | 6 | 3.394 | 0.5657 |
| 160 | 1S5A | Hypothetical protein yesE | 7 | 3.958 | 0.5655 |
| 161 | 1P7O | Phospholipase A2, acidic 2 | 6 | 3.359 | 0.5599 |
| 162 | 1S9C | Peroxisomal multifunctional enzyme type 2 | 6 | 3.334 | 0.5557 |
| 163 | 1TG6 | Putative ATP-dependent Clp protease proteolytic subunit, mitochondrial | 6 | 3.325 | 0.5542 |
| 164 | 2K2Q | Tyrocidine synthetase 3 | 6 | 3.302 | 0.5503 |
| 165 | 2YSD | Membrane-associated guanylate kinase, WW and PDZ domain-containing protein 1 | 6 | 3.297 | 0.5495 |
| 166 | 1GK9 | Penicillin G acylase | 7 | 3.844 | 0.5491 |
| 167 | 2JPE | Nuclear inhibitor of protein phosphatase 1 | 6 | 3.289 | 0.5482 |
| 168 | 2HCB | Chromosomal replication initiator protein dnaA | 7 | 3.836 | 0.548 |
| 169 | 1FIZ | Acrosin | 6 | 3.286 | 0.5477 |
| 170 | 1K44 | Nucleoside diphosphate kinase | 6 | 3.275 | 0.5458 |
| 171 | 2OQD | Phospholipase A2 bothropstoxin-2 | 6 | 3.264 | 0.5441 |
| 172 | 1YIV | Myelin P2 protein | 8 | 4.343 | 0.5429 |
| 173 | 1TZ7 | 4-alpha-glucanotransferase | 6 | 3.255 | 0.5425 |
| 174 | 2A98 | Inositol-trisphosphate 3-kinase C | 8 | 4.331 | 0.5414 |
| 175 | 1Q8B | Uncharacterized protein yjcS | 7 | 3.778 | 0.5397 |
| 176 | 3BRP | C-phycocyanin alpha chain | 7 | 3.774 | 0.5392 |
| 177 | 2DLW | Docking protein 2 | 7 | 3.762 | 0.5375 |
| 178 | 2HH8 | Uncharacterized protein ydfO | 7 | 3.724 | 0.5321 |
| 179 | 2RKJ | Tyrosyl-tRNA synthetase, mitochondrial | 6 | 3.191 | 0.5319 |
| 180 | 1BQ6 | Chalcone synthase 2 | 7 | 3.717 | 0.5309 |
| 181 | 2DAF | IQ and ubiquitin-like domain-containing protein | 7 | 3.7 | 0.5286 |
| 182 | 2CB2 | Sulfur oxygenase/reductase | 6 | 3.172 | 0.5286 |
| 183 | 1YA0 | Protein SMG7 | 7 | 3.688 | 0.5269 |
| 184 | 1DOS | Fructose-bisphosphate aldolase class 2 | 6 | 3.161 | 0.5269 |
| 185 | 1PUJ | Ribosome biogenesis GTPase A | 6 | 3.161 | 0.5268 |
| 186 | 1VEG | NEDD8 ultimate buster 1 | 7 | 3.68 | 0.5257 |
| 187 | 3C72 | Geranylgeranyl transferase type-2 subunit alpha | 6 | 3.154 | 0.5257 |
| 188 | 1VK0 | Uncharacterized protein At5g06450 | 7 | 3.679 | 0.5256 |
| 189 | 3BOM | Hemoglobin subunit alpha-4 | 7 | 3.679 | 0.5255 |
| 190 | 1JV1 | UDP-N-acetylhexosamine pyrophosphorylase | 6 | 3.151 | 0.5252 |
| 191 | 1Z9F | Single-stranded DNA-binding protein | 6 | 3.147 | 0.5245 |
| 192 | 2FRX | Ribosomal RNA small subunit methyltransferase F | 7 | 3.669 | 0.5241 |
| 193 | 3E7O | Mitogen-activated protein kinase 9 | 6 | 3.143 | 0.5238 |
| 194 | 1XGE | Dihydroorotase | 6 | 3.129 | 0.5214 |
| 195 | 2QV6 | GTP cyclohydrolase III | 6 | 3.125 | 0.5209 |
| 196 | 1QIB | 72 kDa type IV collagenase | 8 | 4.166 | 0.5207 |
| 197 | 2HYD | Putative multidrug export ATP-binding/permease protein SAV1866 | 6 | 3.124 | 0.5207 |
| 198 | 1B4U | Protocatechuate 4,5-dioxygenase alpha chain | 6 | 3.11 | 0.5183 |
| 199 | 1JDE | Pyruvate, phosphate dikinase | 6 | 3.105 | 0.5175 |
| 200 | 3DH4 | Sodium/glucose cotransporter | 6 | 3.093 | 0.5156 |
| 201 | 2ZUY | Rhamnogalacturonan lyase yesX | 6 | 3.091 | 0.5152 |
| 202 | 1WXR | Hemoglobin-binding protease hbp | 8 | 4.112 | 0.514 |
| 203 | 2CXQ | Glucose-6-phosphate isomerase | 7 | 3.592 | 0.5131 |
| 204 | 1WZ8 | Enoyl-CoA hydratase | 7 | 3.59 | 0.5129 |
| 205 | 1WI5 | Protein RRP5 homolog | 6 | 3.076 | 0.5127 |
| 206 | 1SPU | Primary amine oxidase | 6 | 3.074 | 0.5124 |
| 207 | 1MG5 | Alcohol dehydrogenase | 8 | 4.096 | 0.512 |
| 208 | 1R19 | Serine-aspartate repeat-containing protein G | 7 | 3.583 | 0.5118 |
| 209 | 1VMD | Methylglyoxal synthase | 7 | 3.577 | 0.511 |
| 210 | 1VAP | Phospholipase A2 | 8 | 4.08 | 0.51 |
| 211 | 1INZ | Epsin-1 | 7 | 3.569 | 0.5098 |
| 212 | 1D06 | Sensor protein fixL | 7 | 3.567 | 0.5096 |
| 213 | 1QE0 | Histidyl-tRNA synthetase | 6 | 3.051 | 0.5085 |
| 214 | 1SGM | Uncharacterized HTH-type transcriptional regulator yxaF | 6 | 3.037 | 0.5062 |
| 215 | 2AU3 | DNA primase | 6 | 3.034 | 0.5057 |
| 216 | 1KMH | ATP synthase subunit alpha, chloroplastic | 6 | 3.021 | 0.5035 |
| 217 | 1UKF | Cysteine protease avirulence protein avrPphB | 6 | 3.013 | 0.5022 |
| 218 | 2CE4 | Superoxide dismutase [Mn] | 6 | 3.013 | 0.5022 |
| 219 | 1K0E | Para-aminobenzoate synthase component 1 | 6 | 3.01 | 0.5017 |
| 220 | 2D2M | Extracellular giant hemoglobin major globin subunit A1 | 6 | 3.003 | 0.5006 |
| 221 | 1VCN | CTP synthase | 8 | 4 | 0.5 |
| 222 | 1N46 | Thyroid hormone receptor beta | 6 | 3 | 0.5 |
| 223 | 1W98 | G1/S-specific cyclin-E1 | 6 | 2.999 | 0.4999 |
| 224 | 1YFM | Fumarate hydratase, mitochondrial | 7 | 3.498 | 0.4997 |
| 225 | 2GNX | UPF0536 protein C12orf66 homolog | 6 | 2.998 | 0.4997 |
| 226 | 1TNO | Protein farnesyltransferase/geranylgeranyltransferase type-1 subunit alpha | 6 | 2.998 | 0.4997 |
| 227 | 1WJT | Transcription elongation factor A protein 3 | 6 | 2.995 | 0.4991 |
| 228 | 1GQ2 | NADP-dependent malic enzyme | 6 | 2.994 | 0.499 |
| 229 | 2OAY | Plasma protease C1 inhibitor | 6 | 2.992 | 0.4987 |
| 230 | 1NJF | DNA polymerase III subunit tau | 6 | 2.992 | 0.4986 |
| 231 | 1SED | Hypothetical protein yhaI | 6 | 2.991 | 0.4985 |
| 232 | 2CZY | Paired amphipathic helix protein Sin3b | 6 | 2.99 | 0.4984 |
| 233 | 1ML8 | Protein yhfA | 6 | 2.989 | 0.4982 |
| 234 | 2AAO | Calcium-dependent protein kinase 1 | 6 | 2.989 | 0.4982 |
| 235 | 1WS1 | Peptide deformylase 1 | 6 | 2.989 | 0.4981 |
| 236 | 1YVW | Phosphoribosyl-ATP pyrophosphatase | 6 | 2.989 | 0.4981 |
| 237 | 2OYZ | UPF0345 protein VPA0057 | 6 | 2.988 | 0.4981 |
| 238 | 1FDY | N-acetylneuraminate lyase | 6 | 2.988 | 0.498 |
| 239 | 2PBE | Aminoglycoside 6-adenylyltransferase | 6 | 2.988 | 0.498 |
| 240 | 2P63 | Cell division control protein 4 | 6 | 2.988 | 0.498 |
| 241 | 1XS8 | Probable Fe(2+)-trafficking protein | 6 | 2.988 | 0.498 |
| 242 | 1QCB | Heat-labile enterotoxin IIB, B chain | 6 | 2.986 | 0.4977 |
| 243 | 1WUK | Periplasmic [NiFe] hydrogenase large subunit | 6 | 2.986 | 0.4976 |
| 244 | 3DM3 | Replication factor A | 6 | 2.985 | 0.4976 |
| 245 | 1CX1 | Endoglucanase C | 6 | 2.984 | 0.4974 |
| 246 | 2QNC | Recombination endonuclease VII | 6 | 2.984 | 0.4973 |
| 247 | 2GK9 | Phosphatidylinositol-5-phosphate 4-kinase type-2 gamma | 6 | 2.984 | 0.4973 |
| 248 | 2EJR | Lysine-specific histone demethylase 1 | 6 | 2.983 | 0.4972 |
| 249 | 1OVM | Indole-3-pyruvate decarboxylase | 6 | 2.983 | 0.4972 |
| 250 | 2VW5 | ATP-dependent molecular chaperone HSP82 | 6 | 2.983 | 0.4971 |
| 251 | 1BH9 | Transcription initiation factor TFIID subunit 13 | 6 | 2.982 | 0.4971 |
| 252 | 1TWU | Uncharacterized protein yycE | 6 | 2.982 | 0.497 |
| 253 | 2IWG | 52 kDa Ro protein | 10 | 4.968 | 0.4968 |
| 254 | 1XVI | Putative mannosyl-3-phosphoglycerate phosphatase | 6 | 2.98 | 0.4967 |
| 255 | 1N83 | Nuclear receptor ROR-alpha | 6 | 2.98 | 0.4967 |
| 256 | 2YT7 | Amyloid beta A4 precursor protein-binding family A member 3 | 6 | 2.98 | 0.4967 |
| 257 | 2I36 | Rhodopsin | 6 | 2.98 | 0.4967 |
| 258 | 1AHJ | Nitrile hydratase subunit alpha | 6 | 2.979 | 0.4965 |
| 259 | 1E+77 | Glucose-6-phosphate 1-dehydrogenase | 6 | 2.978 | 0.4964 |
| 260 | 1A5Z | L-lactate dehydrogenase | 6 | 2.978 | 0.4964 |
| 261 | 2W4O | Calcium/calmodulin-dependent protein kinase type IV | 6 | 2.977 | 0.4962 |
| 262 | 1WH0 | Ubiquitin carboxyl-terminal hydrolase 19 | 6 | 2.976 | 0.496 |
| 263 | 1B8P | Malate dehydrogenase | 6 | 2.976 | 0.496 |
| 264 | 1K20 | Probable manganese-dependent inorganic pyrophosphatase | 6 | 2.976 | 0.496 |
| 265 | 2DMZ | InaD-like protein | 6 | 2.975 | 0.4959 |
| 266 | 1GO3 | DNA-directed RNA polymerase subunit E | 8 | 3.967 | 0.4958 |
| 267 | 2Z2N | Virginiamycin B lyase | 7 | 3.469 | 0.4956 |
| 268 | 1YF2 | Type-1 restriction enzyme MjaXIP specificity protein | 6 | 2.973 | 0.4956 |
| 269 | 2Z3X | Small, acid-soluble spore protein C | 6 | 2.973 | 0.4955 |
| 270 | 1Q23 | Chloramphenicol acetyltransferase | 7 | 3.468 | 0.4954 |
| 271 | 3CUO | Probable HTH-type transcriptional regulator ygaV | 6 | 2.972 | 0.4954 |
| 272 | 2VV5 | Small-conductance mechanosensitive channel | 7 | 3.466 | 0.4952 |
| 273 | 1OMW | Beta-adrenergic receptor kinase 1 | 6 | 2.971 | 0.4952 |
| 274 | 2KA5 | Putative anti-sigma factor antagonist TM_1081 | 6 | 2.971 | 0.4951 |
| 275 | 2VDE | Outer membrane protein tolC | 6 | 2.97 | 0.4951 |
| 276 | 2IF2 | Dephospho-CoA kinase | 6 | 2.97 | 0.495 |
| 277 | 1O7D | Lysosomal alpha-mannosidase | 6 | 2.97 | 0.4949 |
| 278 | 2OWY | Recombination-associated protein rdgC | 6 | 2.968 | 0.4947 |
| 279 | 1MSW | DNA-directed RNA polymerase | 6 | 2.968 | 0.4947 |
| 280 | 1U5H | Citrate lyase beta subunit-like protein | 6 | 2.967 | 0.4945 |
| 281 | 1S6D | Albumin-8 | 8 | 3.955 | 0.4944 |
| 282 | 2VR2 | Dihydropyrimidinase | 6 | 2.966 | 0.4944 |
| 283 | 1JEY | ATP-dependent DNA helicase 2 subunit 1 | 6 | 2.964 | 0.4939 |
| 284 | 1RP8 | Alpha-amylase type A isozyme | 8 | 3.951 | 0.4938 |
| 285 | 2A2F | Exocyst complex component 6 | 8 | 3.95 | 0.4938 |
| 286 | 1N2C | Nitrogenase molybdenum-iron protein alpha chain | 8 | 3.949 | 0.4936 |
| 287 | 1PEV | Actin-interacting protein 1 | 6 | 2.962 | 0.4936 |
| 288 | 1H9T | Fatty acid metabolism regulator protein | 6 | 2.962 | 0.4936 |
| 289 | 1OFH | ATP-dependent hsl protease ATP-binding subunit hslU | 6 | 2.961 | 0.4935 |
| 290 | 1TXK | Glucans biosynthesis protein G | 6 | 2.96 | 0.4933 |
| 291 | 1ASQ | L-ascorbate oxidase | 6 | 2.959 | 0.4932 |
| 292 | 2AFF | Antigen KI-67 | 6 | 2.958 | 0.493 |
| 293 | 1LWD | Isocitrate dehydrogenase [NADP], mitochondrial | 6 | 2.958 | 0.493 |
| 294 | 2HYE | DNA damage-binding protein 1 | 6 | 2.958 | 0.493 |
| 295 | 1SRP | Serralysin | 6 | 2.957 | 0.4928 |
| 296 | 2KBQ | Harmonin | 6 | 2.956 | 0.4927 |
| 297 | 1HJ1 | Estrogen receptor beta | 6 | 2.955 | 0.4924 |
| 298 | 2GZO | UPF0301 protein SO_3346 | 6 | 2.953 | 0.4922 |
| 299 | 2CPT | Vacuolar protein sorting-associated protein 4B | 6 | 2.952 | 0.4921 |
| 300 | 1KU9 | DNA-binding protein MJ1563 | 6 | 2.952 | 0.4921 |


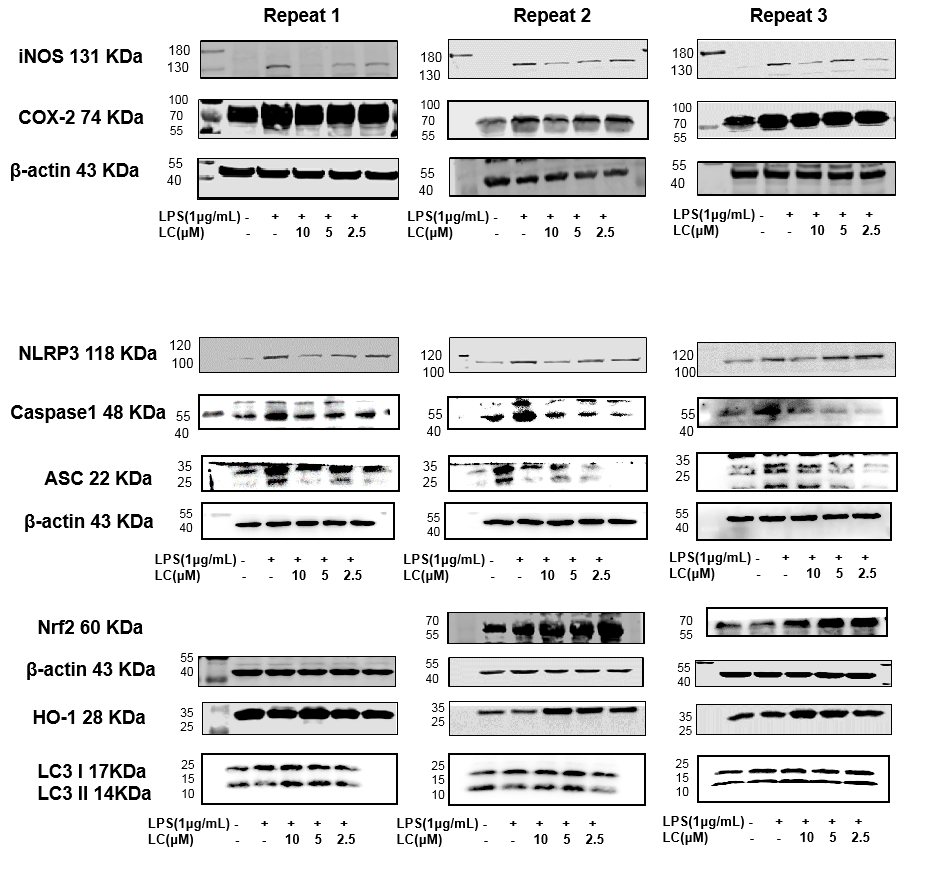


**Figure S5.** Original western blot for three repeats
